# Mapping and Deciphering Neural Codes of NMDA Receptor-Dependent Fear Memory Engrams in the Hippocampus

**DOI:** 10.1371/journal.pone.0079454

**Published:** 2013-11-26

**Authors:** Hongmiao Zhang, Guifen Chen, Hui Kuang, Joe Z. Tsien

**Affiliations:** 1 Brain and Behavior Discovery Institute and Department of Neurology, Medical College of Georgia at Georgia Regents University, Augusta, Georgia, United States of America; 2 Brain Decoding Center, Banna Biomedical Research Institute, Xi-Shuang-Ban-Na Prefecture, Yunnan Province, China; Peking University, China

## Abstract

Mapping and decoding brain activity patterns underlying learning and memory represents both great interest and immense challenge. At present, very little is known regarding many of the very basic questions regarding the neural codes of memory: are fear memories retrieved during the freezing state or non-freezing state of the animals? How do individual memory traces give arise to a holistic, real-time associative memory engram? How are memory codes regulated by synaptic plasticity? Here, by applying high-density electrode arrays and dimensionality-reduction decoding algorithms, we investigate hippocampal CA1 activity patterns of trace fear conditioning memory code in inducible NMDA receptor knockout mice and their control littermates. Our analyses showed that the conditioned tone (CS) and unconditioned foot-shock (US) can evoke hippocampal ensemble responses in control and mutant mice. Yet, temporal formats and contents of CA1 fear memory engrams differ significantly between the genotypes. The mutant mice with disabled NMDA receptor plasticity failed to generate CS-to-US or US-to-CS associative memory traces. Moreover, the mutant CA1 region lacked memory traces for “what at when” information that predicts the timing relationship between the conditioned tone and the foot shock. The degraded associative fear memory engram is further manifested in its lack of intertwined and alternating temporal association between CS and US memory traces that are characteristic to the holistic memory recall in the wild-type animals. Therefore, our study has decoded real-time memory contents, timing relationship between CS and US, and temporal organizing patterns of fear memory engrams and demonstrated how hippocampal memory codes are regulated by NMDA receptor synaptic plasticity.

## Introduction

The major obstacle in understanding of how memory works in the brain is the lack of description of the real-time brain activity patterns and its organizing principles underlying real-time memory process. Most electrophysiological studies examined single unit activity using peri-event based data averaging methods over multiple trials to characterize response or tuning properties of the recording neurons. This data-averaging practice has, unfortunately, led to the loss of crucial information regarding transient activity patterns and fundamental dynamics that underlies real-time memory code. From a structural perspective, memory is believed to be the storage of acquired information in a form of synaptic connectivity patterns via by NMDA receptor-dependent synaptic plasticity [1]–[7]. Yet, from a temporal dynamic perspective, memory traces are real-time transient activity patterns at the moment when the stored information is re-emerged by recall [8], [9]. Information encoding different memory traces evolves dynamically from a moment to moment as the brain retrieves them. Currently, little is known regarding the real-time brain activity patterns of associative memories and what real-time memory engram looks like and how they are organized. There is an emerging interest in applying large-scale neural recording and powerful mathematical decoding approaches to seek out brain activity patterns or brain activity maps hidden inside the large datasets [10]–[13].

Extensive studies have demonstrated that neurons in the hippocampus encode spatial information about animals’ location, categorical information about people and items, and timing relationships [14]–[21], yet the hippocampus is also known to play a central role in trace and contextual fear memories. Fear memory is associative in nature, and fear memory-associated behaviors are well defined and widely studied across a variety of animal species. A wide range of lesion studies on mice, rats, and primates show that trace fear conditioning, especially with longer trace interval, requires the structural integrity of the hippocampus and related circuits [22]–[30]. In rodents, fear memory can be conveniently assessed by measuring the amount of freezing [23]–[25], startle responses [31], [32], or changes in heart rates and heart rate variability [33]. Although behavioral changes indicate the formation of new memories, many mnemonic thoughts and precise contents of memory in animals remains internal and largely inaccessible to outside observers. It has been suggested that fear memory may contain information about CS, US, environmental context, self-location, and the time relationship (or time trace interval) between hearing the tone and expected arrival of foot-shock, etc. [23]–[29], [34]–[36]. However, by assessing freezing behavior from outside, such detailed information generated in the memory circuits can only be inferred. At present, very little is known regarding many very basic questions such as: are fear memory traces retrieved during the freezing state or non-freezing state of the animals? How can different memory contents or traces come together to generate a holistic memory engram in real-time? What are the temporal organizing principles underlying associative memory engram? How fast can memory traces be retrieved in the mouse hippocampus? In seeking the answers to these fundamental questions, in the present study we combine large-scale decoding technologies with inducible NMDA receptor knockout approach to map out hippocampal activity patterns of trace and contextual fear memory traces in the CA1 region. We provide the first detailed description about how the content and temporal dynamics of real-time memory engrams are organized by the NMDA receptor-mediated synaptic plasticity.

## Results

### CA1 ensemble representations for conditioned and unconditioned stimuli

**Figure 1:**
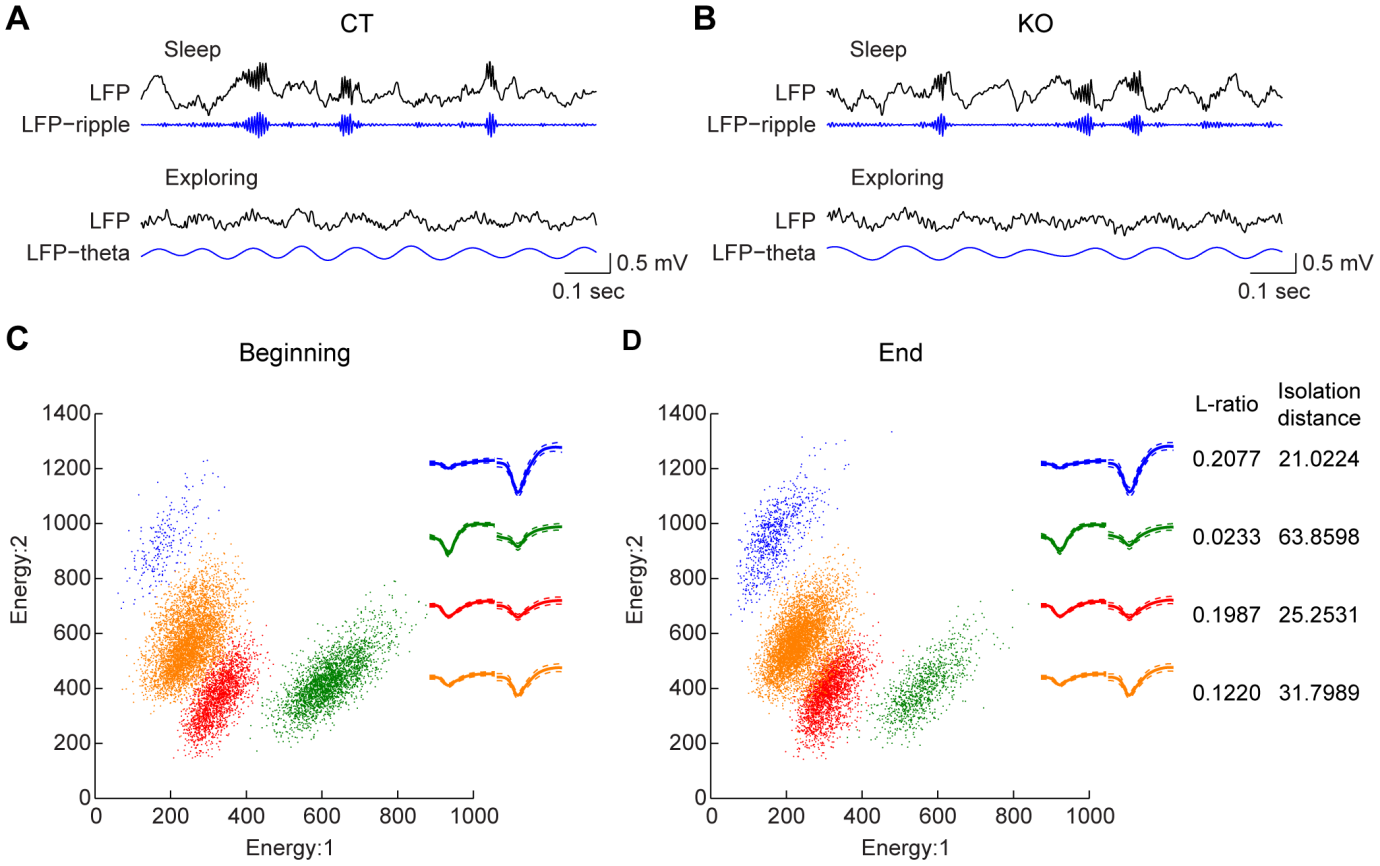
Recording of single units and local field potentials in the hippocampal CA1 region of freely behaving mice. (**A**) The characteristic oscillations confirm the recording happened in the hippocampal CA1 region of a representative control mouse. The top panel shows a representative channel of local field potential (LFP) recorded during sleep, and the filtered LFP shows high-frequency ripple (100–250 Hz). The lower panels show the raw LFP and LFP_theta (4–12 Hz) recorded when the animal was active exploring. Scale bar: 0.5 mV and 0.1 sec. (**B**) Sharp wave associated ripple (top panel) and theta oscillations (bottom panel) were also observed in the CA1 of the knockout mouse hippocampus. (**C-D**) Automatic spike sorting was performed with KlustaKwik method and followed by manual cutting and merging in MClust program. Spike clusters for a typical stereotrode were shown with the energy of the two channels of a stereotrode. The stereotrode waveforms of each unit are shown in the inserts. Stable recordings were confirmed as judged by the distribution of spike clusters and spike waveforms of each individual unit at the beginning (**C**) and end (**D**) of the recording whole session. L-ratio and Isolation distances were used to quantitatively measure the quality of sorted units. The L-ratios of the four units shown here were 0.2077, 0.0233, 0.1987 and 0.1220, respectively, and the Isolation distances were 21.0224, 63.8598, 25.2531 and 31.7989, respectively.

**Figure 2:**
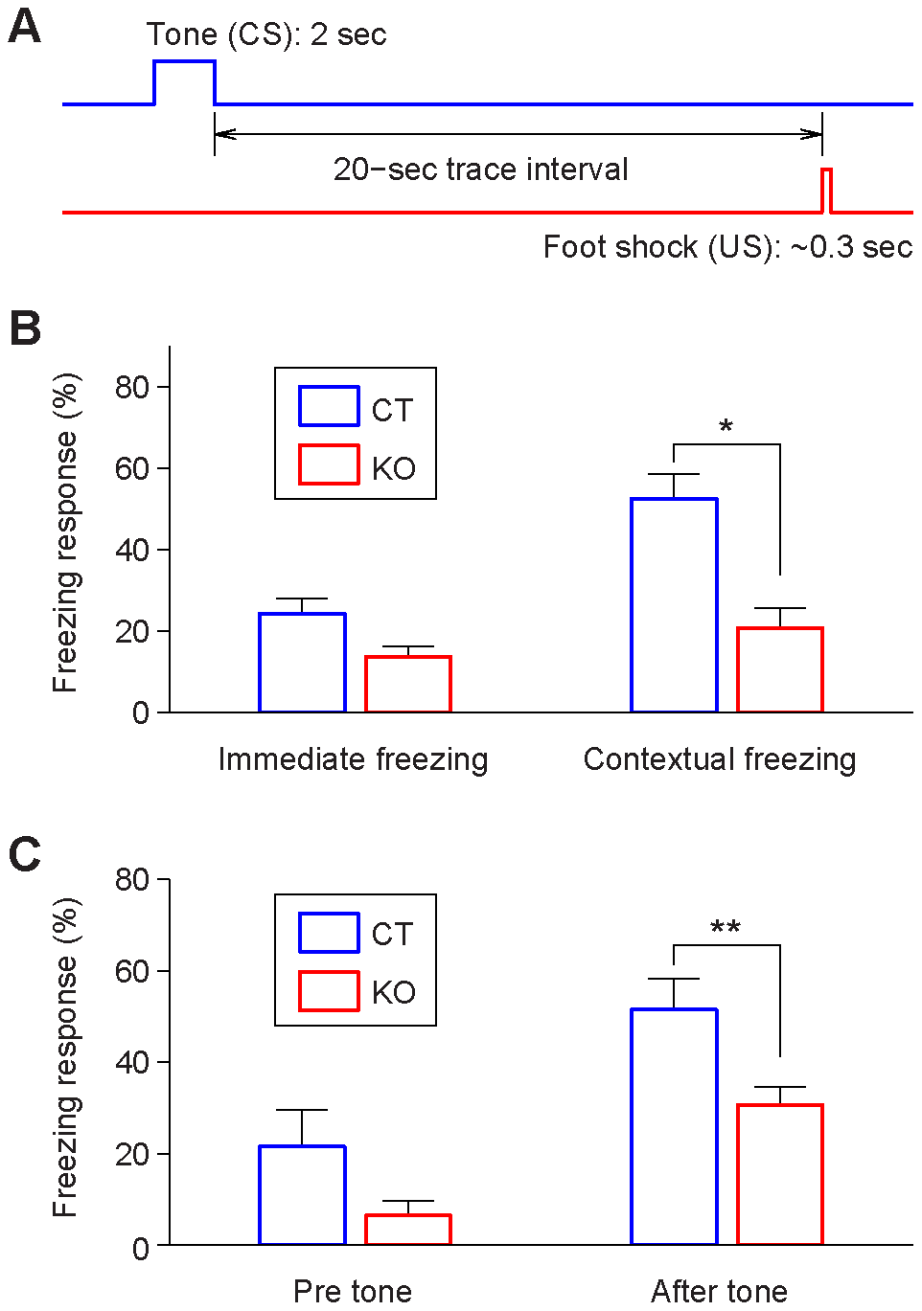
Behavior performances in fear conditioning. (**A**) Illustration of tone-shock trace fear conditioned memory. A 2-sec neutral tone precedes a mild foot-shock (0.3 sec) with a 20-sec time trace interval. Seven pairings were given. (**B**) Immediate freezing during learning and contextual freezing during 1-hr contextual memory recall. There was a significant difference in contextual freezing between the control (*n* = 4, 52%±6%) and mutant mice (*n = *5, 21%±5%). Error bars represent SEM; *p<0.05. (**C**) Impaired trace fear retention in the mutant group as compared to the control group. Freezing prior to recall tone and after the tone at 1-hr trace recall in the control and mutant mice. The tone was presented for seven times (trials) with a 1–3 min random time interval. There was a significant difference in tone-induced freezing between control (51%±7%) and knockout mice (31%±4%), **p<0.01.

We implanted 128-channel electrodes into mouse hippocampal CA1 regions and recorded large numbers of single units from both control and knockout mice. The location of electrodes in the CA1 was initially assessed by the presence of characteristic oscillations sharp-wave associated ripples and theta (Figure 1), and then confirmed by post-recording histology. We recorded a total of 1147 CA1 units from five control mice and 1153 CA1 units from five inducible- and forebrain-specific NMDA receptor knockout mice. We used the trace fear conditioning paradigm to train both the inducible knockout mice and their control littermates (5–6 month old males). The trace conditioning contained a neutral tone (CS, 2 sec of 2.8 kHz pure tone at 85 dB) which was followed by a delayed mild foot shock (US, 0.75 mA, 0.3 sec) with a fixed 20 seconds “trace” time interval between CS and US (Figure 2A). Conditioning of this long-duration time trace memory is highly sensitive to hippocampal-lesion [23]–[29], [34], [35], and the paradigm permits closer examination of various memories such as the memories for the conditioned tone, shock event, their causal relationship, and time information for the tone and shock intervals, etc. [36]. To facilitate the physiological identification of CS and/or US response units in the CA1 of the hippocampus, we used seven repetitions of CS/US pairing protocol which produced strong contextual and trace fear memories in the control mice as assessed in 1-hr retention tests. We also examined the genetic effects of forebrain-specific NMDA receptor deletion on trace fear conditioning by feeding the inducible knockout mice with doxycycline 5 days prior to fear conditioning. As expected, dox-induced NMDA receptor knockout caused significant behavioral impairment in learning trace fear conditioning as revealed by reduced contextual freezing (Figure 2B, Student’s *t* test *p* <0.05) and trace retention freezing (Figure 2C, Student’s *t* test *p* <0.01).

**Figure 3:**
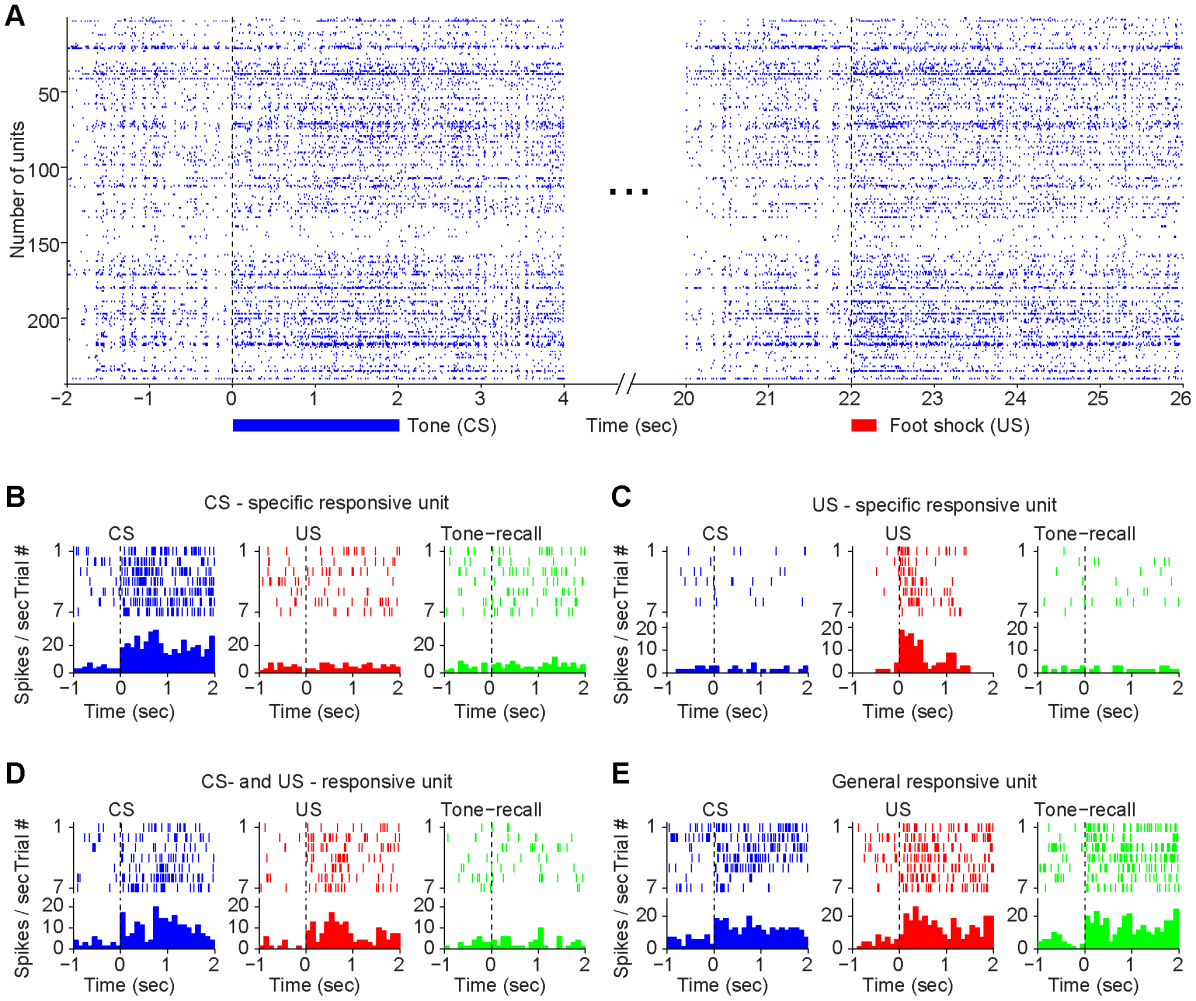
CA1 responses to fear conditioning. (**A**) Spike rasters of simultaneously recorded 243 CA1 units from a control mouse in response to the conditioned tone and foot-shock during training. (**B**) A representative unit only responded to CS during learning. (**C**) A representative unit responded only to CS-paired foot shock during learning. (**D**) A representative unit responded to both CS and tone-paired US, but not to CS during recall. (**E**) A representative unit responded to CS and US during pairing as well as to recall tone. Within each panel, upper subplot is peri-event raster; each short vertical tick presents a spike. Spike activities are aligned at the time when stimuli were delivered. Lower subplot shows histogram calculated with 100 msec temporal windows.

**Figure 4:**
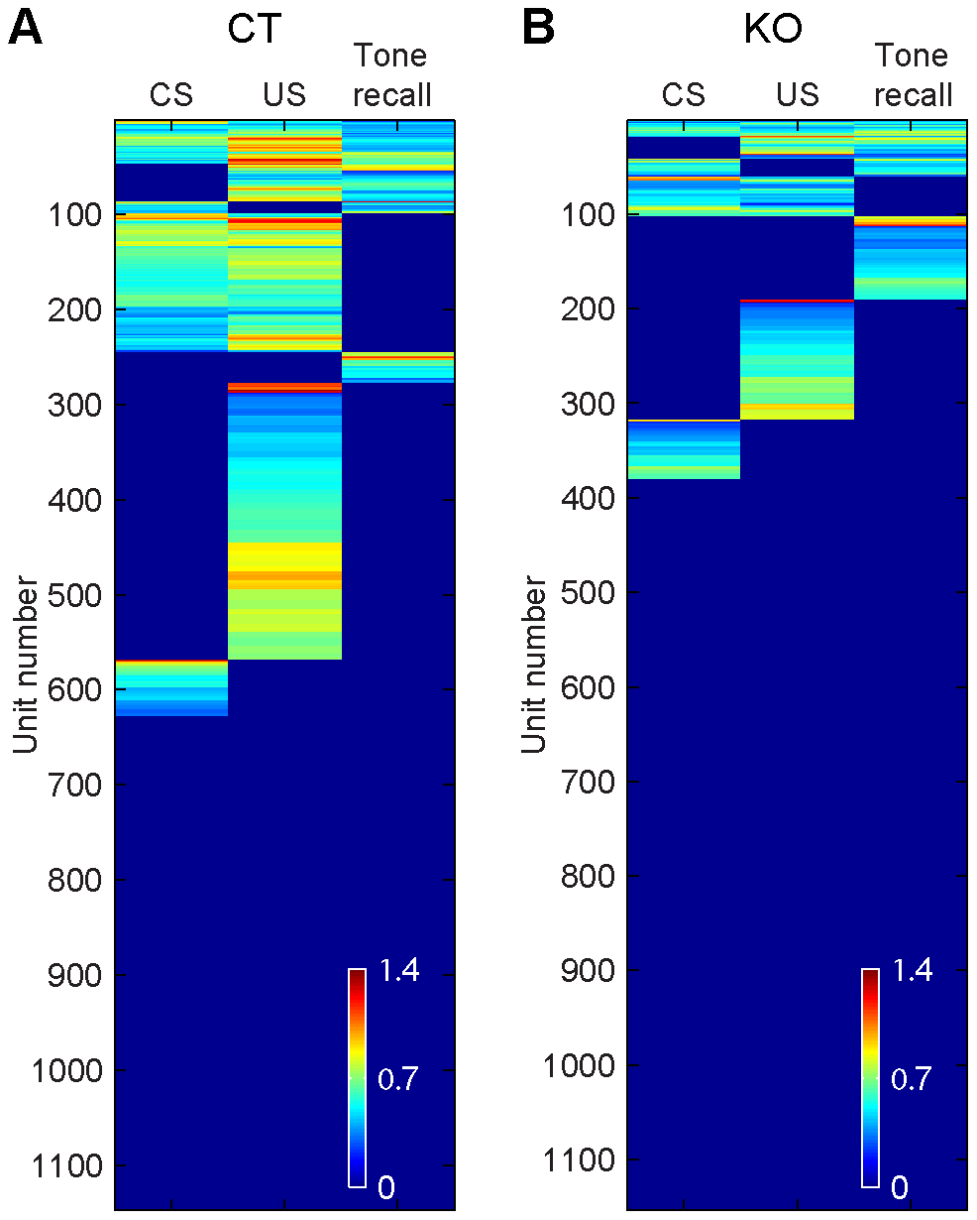
Overview of fear conditioning responses of recorded hippocampal CA1 cell populations. (**A**) Hierarchical clustering method revealed categorical and combinatorial response patterns in recorded CA1 units from the control mice. Nonresponsive units and units responded to CS and US during learning and to the conditioned tone at recall were listed vertically (a total of 1147 units from 5 five control mice). (**B**) Global responses of all recorded CA1 units from the knockout mice (a total of 1153 units from 5 five knockout mice). Color scale bars indicate the logarithm transformed responsiveness of hippocampal units averaged over seven trials (see Methods).

Our 128-channel electrode arrays [36]–[39] permitted us to record simultaneously, on average, the activity of 229±10 units per control mouse or 231±9 units per mutant mice from the CA1 region of the hippocampus bilaterally as these animals underwent the acquisition and 1-hr retention tests. CS and US stimulation evoked firing changes in many of the recorded CA1 units (Figure 3A). To characterize responsiveness of the recorded CA1 units, we conducted peri-event spike raster and histogram analyses by averaging neural responses over seven CS or US during learning, or the conditioned tone during recall trials using the stimulus onset as time zero. Peri-event histograms showed that the conditioned tone stimulus (CS) and foot-shock (US) evoked significant changes in firing of some CA1 units during learning. Some units fired selectively to the CS only during learning (Figure 3B), whereas other units responded only to US (Figure 3C). Moreover, some units changed their firings to both CS and US during learning, but not during tone recall (Figure 3D). In addition, we found a certain percentage of the units would respond to both CS and US during learning and the recall tone during retention tests (Figure 3E). By using the hierarchical clustering analysis [38], [40], we examined and compared that the entire CA1 population activity responsiveness between the genotypes (Figure 4). The overview of the all datasets suggest that the CA1 populations in the control mice exhibited categorical and combinatorial coding patterns in responding to fear conditioning, ranging from broadly associative response units (listed on the top of the plot) to a set of specific response units (listed at the lower portion of the plot) (Figure 4A). Similarly, the mutant mice also showed categorical and combinatorial patterns, but with a lower percentage of responsive cells, especially those combinatorially associative units (Figure 4B). As a whole, approximately 22.9% (263 out of 1147 units) of the recorded units from the control mice and 12.3% (142 out of 1153 units) of the recorded units from knockout mice responded to CS, whereas 45.9% (523 out of 1147) of the recorded units from controls and 18.2% (210 out of 1153) of units from mutants reacted to CS-paired foot-shock. Among CS-responding units, 5.1% (59 out of 1147 units) was specific to CS (not responding to US or Tone at recall) in the controls, whereas 5.5% (63 out of 1153 units) was specific to CS in the mutants. The total percentages of units belonging to the specific groups (i.e. combining CS-specific, CS-paired US-specific, and recall tone-specific units) from the wild-type and mutant CA1 populations were 33.4% and 24.1%, respectively.

Because associative binding of distinct information is a hallmark feature of Pavlovian fear conditioning, we examined more closely the associativeness of the CA1 responding units to both tone and shock. We found that controls had a total 192 units (out of 1147) responded to both CS and US during learning (16.7%) (Figure 4A), whereas the mutants had only 60 units exhibited such associative responses (5.2%) (Figure 4B), reflecting a reduced associative binding between the tone and foot-shock in mutant CA1. Furthermore, the control group had a total of 46 units out of 1147 (4.0%), exhibiting significant responses to both CS and US during learning as well as to the conditioned tone at trace retention tests (Figure 4A). By comparison, the mutant mice had only 18 such units responding to all three stimuli (1.6%) (Figure 4B). Interestingly, the tone at trace retention test resulted in 11.4% (131 out of 1147) of the control units changing their firings (Figure 4A), whereas the mutant mice had 12.8% (148 out of 1153) of the units responded to the recall tone (Figure 4B). This suggests that although the similar percentage of cells in mutant mice responded to tone during the retention tests, many of these units did not participate in CS and/or US learning.

### NMDA receptor knockout prevented the formation of real-time associative memory traces

**Figure 5:**
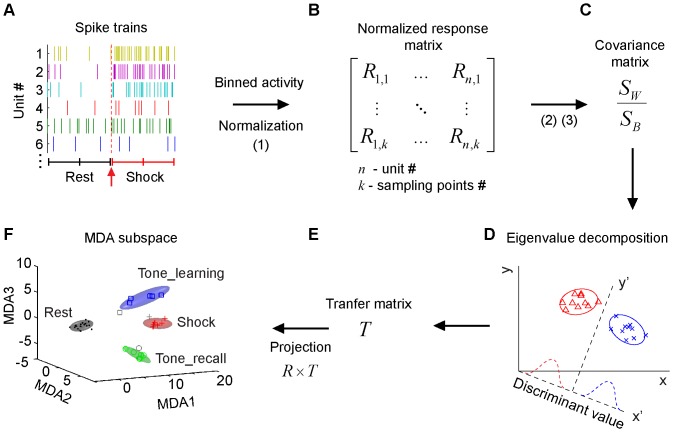
Schematic illustration of Multiple Discriminant analysis (MDA) method for projecting neural ensemble responses to pattern encoding subspaces. (**A**) From spike trains of CA1 units, firing rates in two 250 msec windows after stimuli presentation was extracted. (**B**) After binned and normalized, firing rates were transformed to measure to neural responses. The matrix of normalized response from CA1 units corresponding to the sampling points (all repetitions of CS or US or Rest) was obtained. (**C**) The covariance matrix can be determined by between-class matrix and within-class matrix, which were obtained from population responses matrix. (**D**) The discriminant projection vectors are determined by the eigenvalue decomposition of covariance matrix. (**E**) Transfer matrix was constructed with the corresponding eigenvectors as columns and were sorted in the descend order of the eigenvalues. (**F**) Neural ensemble responses are then projected to form event- and resting state- clusters in MDA pattern encoding subspaces by transfer matrix. The top three most discriminant subspaces (MDA1-3) are plotted for intuitive visualization. A sliding-window technique can be further applied to calculate transient ensemble states of neural activity (using 20 millisecond steps), thereby tracking dynamic evolution of ensemble trajectories in time throughout the entire recording experiments.

**Figure 6:**
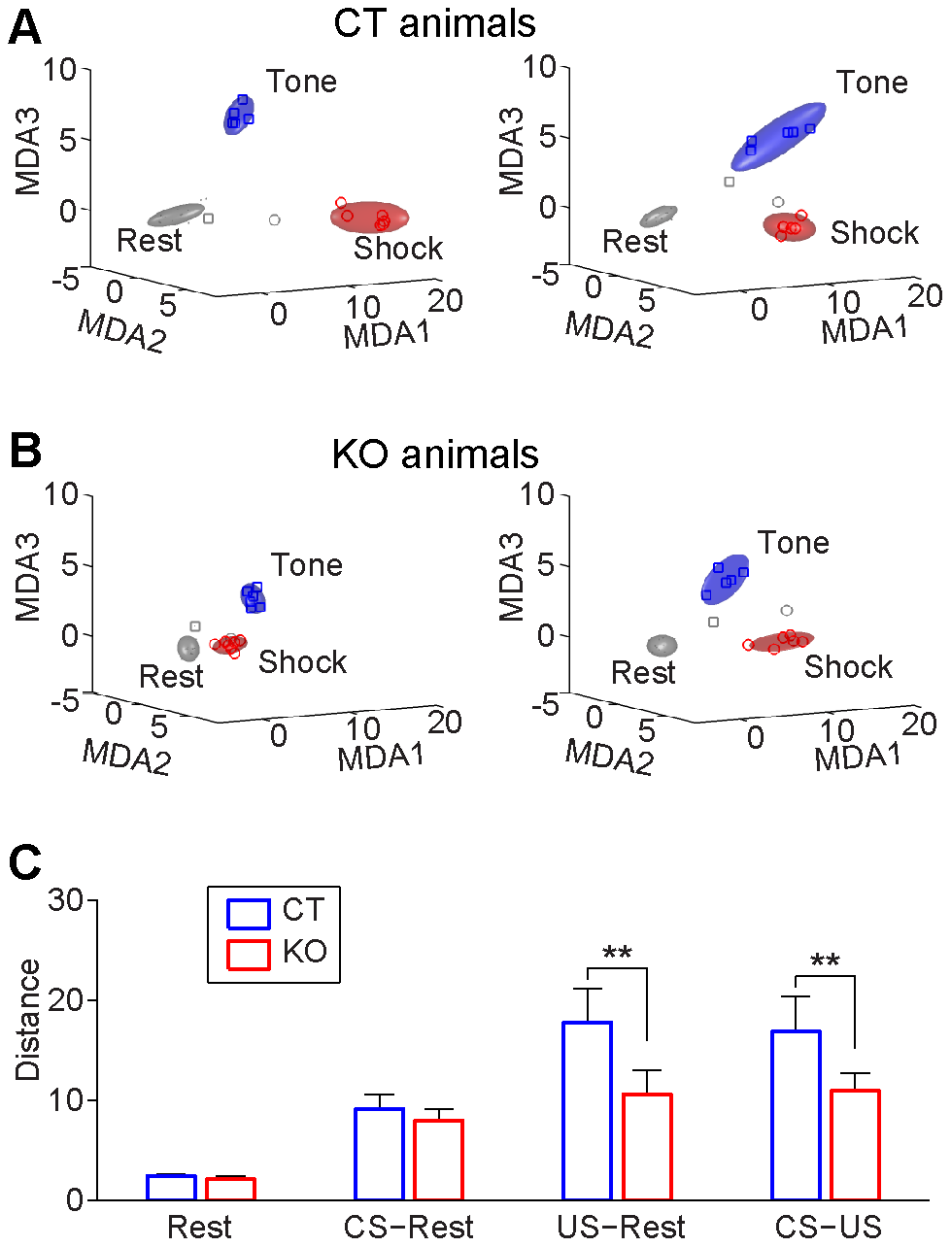
CA1 ensemble representations for the resting state, CS, and CS-paired US states in control and knockout mice. (**A**) MDA projection of CA1 ensemble firing patterns in two representative control mice (left and right plots, respectively). Ensemble activity patterns form distinct states for the rest period prior to CS (dots, grey ellipsoid), conditioned tone state (CS, square, blue ellipsoid) and CS-paired foot-shock state (US, circle, red ellipsoid). The top three most discriminant subspaces were plotted to show the encoding patterns revealed by MDA-based dimensionality-reduction method. (**B**) MDA projection of CA1 ensemble firing patterns from two representative knockout mice (left and right plots, respectively). (**C**) Discriminant distances between points within the Rest cluster, separation distances between the Rest and CS or US clusters, or between the CS and US clusters. Distances were calculated by averaging the mean distances between point to point belonging to different clusters or the same cluster (Rest) over animals. (Student *t-*test, **p<0.01). The knockout mice can still form CS and US ensemble representation, but exhibited lower pattern separation at the CA1 level.

Peri-event spike raster histogram methods, by averaging unit responses over trials, intrinsically limit the data analyses to the time points of CS and US presentations. To investigate real-time memory trace formation at various time points within any given trial, we employed *Multiple Discriminant Analysis* (MDA) to obtain statistical pattern classifications of neural ensemble activities representing distinct stimulus categories (see the schematic outline of MDA method and steps for obtaining dimensionality reduction-based pattern classification as described in Figure 5). The MDA method provides not only quantitative classification of neural ensemble patterns but also intuitive visualization of these patterns in encoding subspaces [36]–[38], [40], [41]. CA1 ensemble activity prior to CS stimulus presentation formed the Rest ellipsoid, whereas the activity during CS or CS-paired foot shock formed the CS tone ellipsoid and US shock ellipsoid, respectively (Figure 6A). Interestingly, CA1 ensemble responses from both control and mutant mice produced reliable classifications for the rest, CS, and US ensemble patterns (Figure 6A and 6B). However, the discriminant distances between the Rest and US ellipsoids or between the CS (Tone) and US ellipsoids in the mutant group are significantly smaller than those of the control littermates (Figure 6C, Student’s *t* test *p*<0.01).

**Figure 7:**
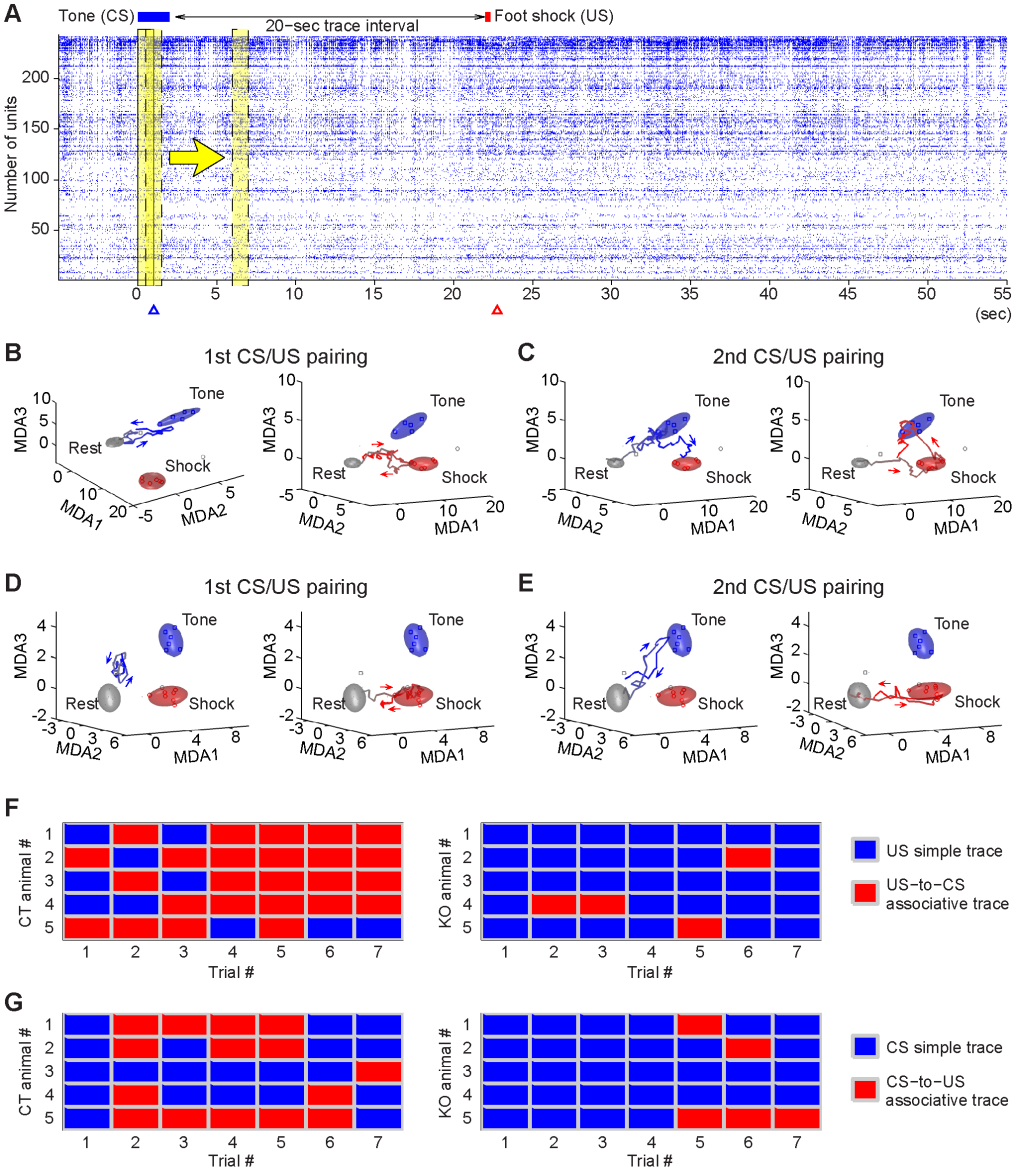
Real-time CS and US simple traces and US-to-CS or CS-to-US associative traces during learning. (**A**) Sixty-sec spike raster of 243 simultaneously recorded CA1 units from a control mouse during the first CS/US pairing. Yellow strips indicate the sliding MDA window. (**B**) MDA dimensionality-reduction statistical projection method show distinct CA1 ensemble patterns (as ellipsoid clusters) represent the Rest (grey), CS (blue) and US (red) activated states. The boundaries of each ellipsoid reflect the 2σ boundaries with Gaussian distributions in the MDA space. Each dot within an ellipsoid shown the MDA subspaces is a statistical result for the ensemble of the simultaneously recorded units from a single trial. The sliding-window method provides the 20-msec resolution of continuous transient ensemble trajectories (or dynamic traces) in response to the CS or US events. A simple CS and US trace during the trial were shown, respectively. The arrows indicate the moving directions of the ensemble trajectories. (**C**) A CS-to-US associative trace (left) and a US-to-CS associative trace (right) were elicited by the tone or foot shock, respectively, at the 2nd CS/US pairing in the same control mouse. (**D**) A simple CS trace (left) and US trace (right) in the mutant mouse at the 1st trial. (**E**) A robust CS or US trace was produced by tone or foot shock, respectively, during the 2nd CS/US pairing trial in the same knockout animal. (**F**) The occurrences of US-to-CS associative traces triggered by the CS-paired foot shock in all seven trials from the five control mice (left panel) and five knockout mice (right panel). The blue rectangles indicate that the simple US traces produced by paired foot-shock, whereas the red rectangles indicate the US-to-CS associative traces were elicited by the US presentation. (**G**) The occurrences of CS-to-US associative traces triggered by the tone in all seven trials from the five control mice (left matrix) and five knockout mice (right matrix). The blue rectangles indicate that the simple CS traces produced by paired CS, whereas the red rectangles indicate the CS-to-US associative traces were elicited by the CS presentation during learning.

By further coupling MDA with the sliding-window method (using 20 millisecond steps) (Figure 7A), we investigated dynamic patterns of CA1 cell population ensembles during the learning phase. We observed that in response to the first tone (before the first paired foot shock arrived), CA1 cell population from the control animal produced a CS ensemble trajectory from the Rest ellipsoid (Figure 7B, left subplot), whereas the CS-paired US (20 seconds after the CS) elicited US ensemble trajectory (Figure 7B, right subplot). Interestingly, as CS/US pairings were repeated over learning trials, the subsequent conditioned tone produced either robust CS simple traces or CS-to-US associative traces (Figure 7C left subplot). Moreover, the CS-paired foot-shock frequently evoked US-to-CS associative trajectories instead of simple US trajectories (Figure 7C right subplot).

By comparison, fear conditioning in knockout mice still triggered simple CS or simple US trajectories at the first CS/US representation (Figure 7D). However, the repeated CS/US pairings over trials rarely led to US-to-CS associative trajectories or CS-to-US associative trajectories (Figure 7E). Of five recorded control mice over seven learning trials, foot shock-induced US-to-CS associative traces occurred predominantly, for 25 times out of a total of 35 CS-paired US presentations (Figure 7F, see red rectangles in the left panel). In contrast, only four such associative traces were observed in the knockout mice (Figure 7F, right panel). Similarly, while CS-induced CS-to-US associative traces (red rectangles) were frequently observed in control mice (15 times out of a total of 35 trials) (Figure 7G, left panel), only 5 such trajectories were seen in the mutant mice (Figure 7G, right panel). Therefore, these results demonstrate that disabling of NMDA receptor function prior to learning impaired the formation of real-time associative ensemble traces in the hippocampus.

### On-line reverberations during the learning phase are diminished by NMDA receptor knockout

**Figure 8:**
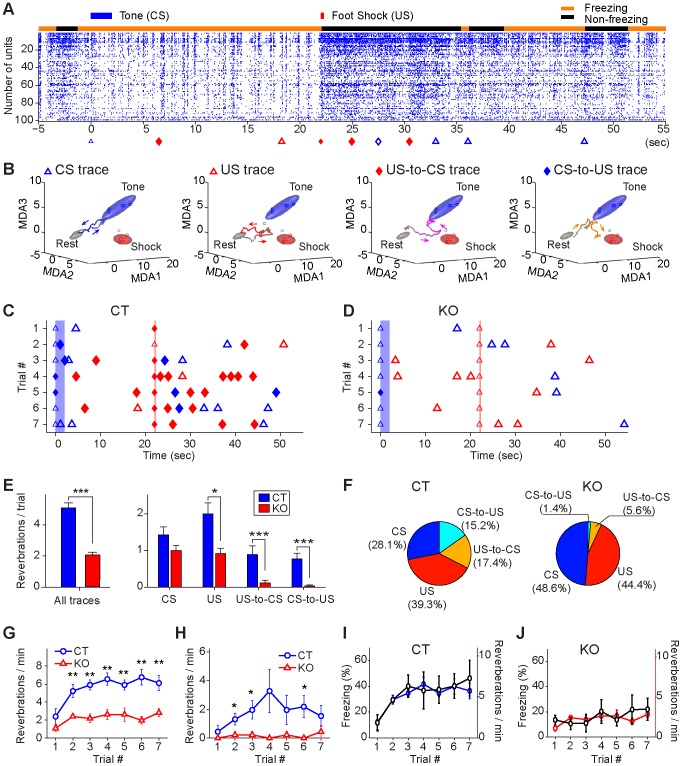
Immediate on-line reverberations of newly acquired CA1 traces during learning phase. (**A)** Reverberation of real-time ensemble traces during the CS/US pairings. The black bar on the top of the spike raster (100 simultaneously recorded CA1 units were selected for illustration) indicates the non-freezing state, whereas the orange bar indicates the freezing state of the animal during learning. The reverberation of various ensemble traces is shown at the bottom of the raster: CS trace, blue triangle; US trace, red triangle; US-to-CS associative trace, red diamond; CS-to-US associative trace, blue diamond. (**B**) Examples of reverberated CS, US, US-to-CS, and CS-to-US traces during the learning phase. (**C**) Increased on-line reverberations of various traces in a representative control mouse over seven CS-US pairing trials. CS and US stimulation time windows are shown by the blue bar and the vertical line, respectively. (**D**) Example of sporadic on-line reverberations from a knockout mouse over the seven pairing trials, with associative traces rarely showing reverberation. (**E**) Average on-line reverberations per trial in control and mutant groups (Wilcoxon rank sum test, ***p<0.001). Left plot for total reverberation numbers in control and mutant groups. Right panel for each type of reverberated traces (genotype difference: Wilcoxon rank sum test, *p<0.05, ***p<0.001). (**F**) Compositions of reverberation numbers for each trace type (CS, US, US-to-CS and CS-to-US) in control and mutant groups. (**G**) Trial-dependent increases in on-line reverberation of both simple and associative traces in the control but not in the mutant groups (*n* = 5 for each group). Student *t*-test, **p<0.01. (**H**) Increased on-line reverberation of associative traces (CS-to-US and US-to-CS traces combined) in the control, but not mutant mice (*n* = 5 for each group). Student *t*-test, *p<0.05, error bars represent SEM. (**I**) The increase in the numbers of learning pattern reverberations was correlated with the increase in the amounts of immediate freezing during this learning phase in control mice (*r* = 0.84, *p<*0.05). (**J**) Lower level of freezing responses and learning pattern reverberations in knockout mice (*r* = 0.28, *p* > 0.05).

To further investigate CA1 activity dynamics during the learning phase, we used the MDA/sliding-window techniques and scanned through the spike rasters during each of seven CS/US pairing trials (60 second) (Figure 8A). This sliding-window technique revealed that the CA1 cell population of control mice produced immediate *on-line reverberations* (we used this term as supposed to *off-line replay* after the training or during sleep). Examples of the time points at which CA1 ensemble trace reverberated are marked by triangles and diamonds shown in Figure 8A. The reverberated traces included the CS simple traces (blue triangles), US simple traces (red triangles), and US-to-CS associative traces (red diamonds) as well as CS-to-US associative traces (blue diamonds) (Figure 8B). Moreover, numbers of trace reverberations in control mice became more prevalent as CS/US pairings were repeated (Figure 8C). By comparison, inducible knockout of the NMDA receptors had drastically reduced reverberation of CS or US traces during the learning phase (Figure 8D). Statistical analyses show that the control mice exhibited much higher amount of total reverberations in comparison to the mutant mice (Figure 8E, left plot, Student’s *t* test *p*<0.001). The differences in reverberation were observed when we analyzed subclasses of various trace types, such as CS-paired simple US traces, US-to-CS associative traces, and CS-to-US associative traces (Figure 8E, right plot, Student’s *t* test *p*<0.05, *p*<0.001, *p*<0.001 respectively). More importantly, the compositions of reverberated trace types are also different between the genotypes. Trace reverberations in the control mice consisted of 67.4% simple traces (28.1% simple CS, 39.3% simple US traces) and 32.6% associative traces (15.2% CS-to-US traces, 17.4% US-to-CS traces) (Figure 8F, left pie chart). For the knockout mice, trace reverberation was not only lower in numbers but also overwhelmingly belonged to simple US or CS traces (93%) (Figure 8F, right pie chart).

The group data analyses further revealed that while the control group had more trace reverberations especially over initial multiple trials, the mutant group did not exhibit any increase (Figure 8G). Furthermore, CS/US pairing repetition over trials seemed to preferentially increase reverberations of associative traces in the control mice (Figure 8H, blue line). By contrast, the mutant group had little reverberation (Figure 8H, red line).

In fear conditioning literature, immediate freezing is often used as an indication for learning. Therefore, we examined how ensemble trace reverberation reflects this form of behavioral learning. We found that the reverberation in the control mice is correlated nicely with the amount of immediate freezing during this learning phase (*r* = 0.84, *p<*0.05) (Figure 8I), whereas the mutant mice had low reverberations and low freezing (Figure 8J). This correlation provides additional support for the validity of the MDA-based decoding approach.

**Figure 9:**
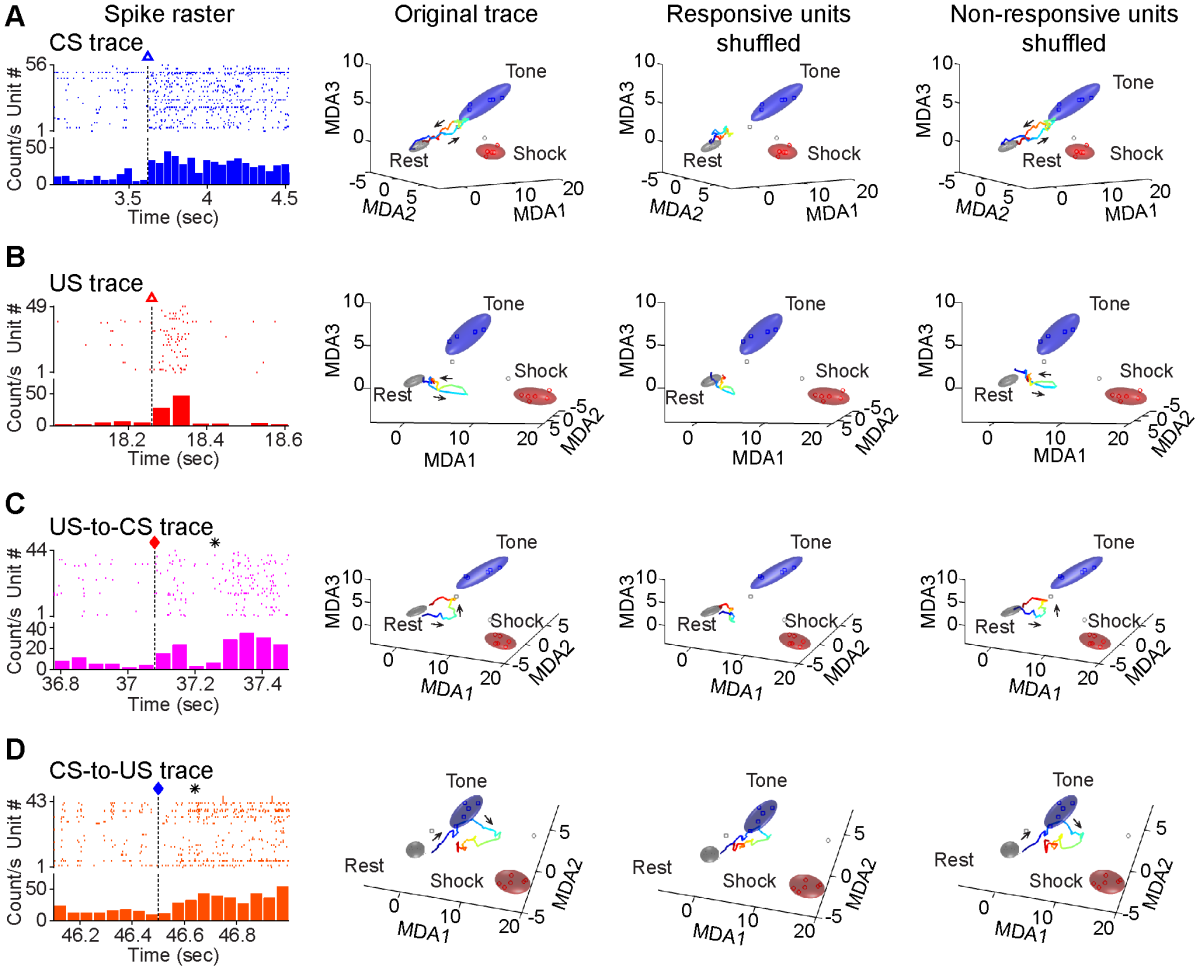
Activated ensemble CA1 units encoding simple or associative traces during reverberations, and their contributions to real-time ensemble trajectories described in MDA encoding subspaces. (**A**) Spike raster of 56 CS-responding units corresponding to a CS trace reverberation (left). This particular reverberated CS ensemble trace is shown in MDA plot prior to spike training shuffle (left MDA plot); selective degradation of this CS trace after shuffling of these units’ spikes (middle MDA plot), but not affected by shuffling of randomly selected 56 non-responsive units (right MDA plot). (**B**) Spike raster of 49 US responsive units corresponding to a US trace reverberation. This reverberated trace is shown in MDA plot prior to shuffle (left MDA plot); selective degradation of this US trace after shuffling of these units’ spikes (middle MDA plot), but not affected by shuffling of randomly selected 49 non-responsive units (right MDA plot). (**C**) Spike raster of 44 responsive units corresponding to a US-to-CS associative trace reverberation. This reverberated associative trace is shown in MDA plot prior to shuffle (left MDA plot); selective degradation of this trace after shuffling of these units’ spikes (middle MDA plot), but not affected by shuffling of randomly selected 44 non-responsive units (right MDA plot). (**D**) Spike raster of 43 responsive units corresponding to a CS-to-US associative trace reverberation. This reverberated trace is shown in MDA plot prior to shuffle (left MDA plot); selective degradation of this trace after shuffling of these units’ spikes (middle MDA plot), but not affected by shuffling of randomly selected 49 non-responsive units (right MDA plot). Arrows show the moving direction of the trajectories.

To identify the neurons that underlie these simple and associative trajectories, we used the time points of reverberated traces revealed by MDA methods as time zero for a new round of peri-event raster and histogram analyses. Indeed, we identified many CA1 units which responded at these time points (Figure 9). These distinct units, through the temporal co-spiking dynamics, collectively produced robust real-time ensemble activity peaks for CS (Figure 9A), US (Figure 9B), US-to-CS (Figure 9C), or CS-to-US (Figure 9D), respectively (raster plots on the left column of Figure 9A-D). To further evaluate these units’ contribution to real-time ensemble trajectories, we employed spike shuffle techniques to examine their trajectories in the MDA subspaces. Indeed, when these responsive units were shuffled, each corresponding trajectory was greatly diminished in comparison to that of before shuffle (Figure 9, prior to shuffle MDA plot on the left, after shuffle MDA plot in the middle). To rule out the overall non-specific effects of random shuffling of spikes, we shuffled an equal number of non-responsive units and found that it did not produced any significant effect on these trajectories (Figure 9, right MDA plot column). Collectively, these analyses strongly suggest that these dynamic trajectories were the results of collective co-firing changes of these perspective units.

### Real-time hippocampal memory traces, including “what at when” memory trace, during trace fear retention test

**Figure 10:**
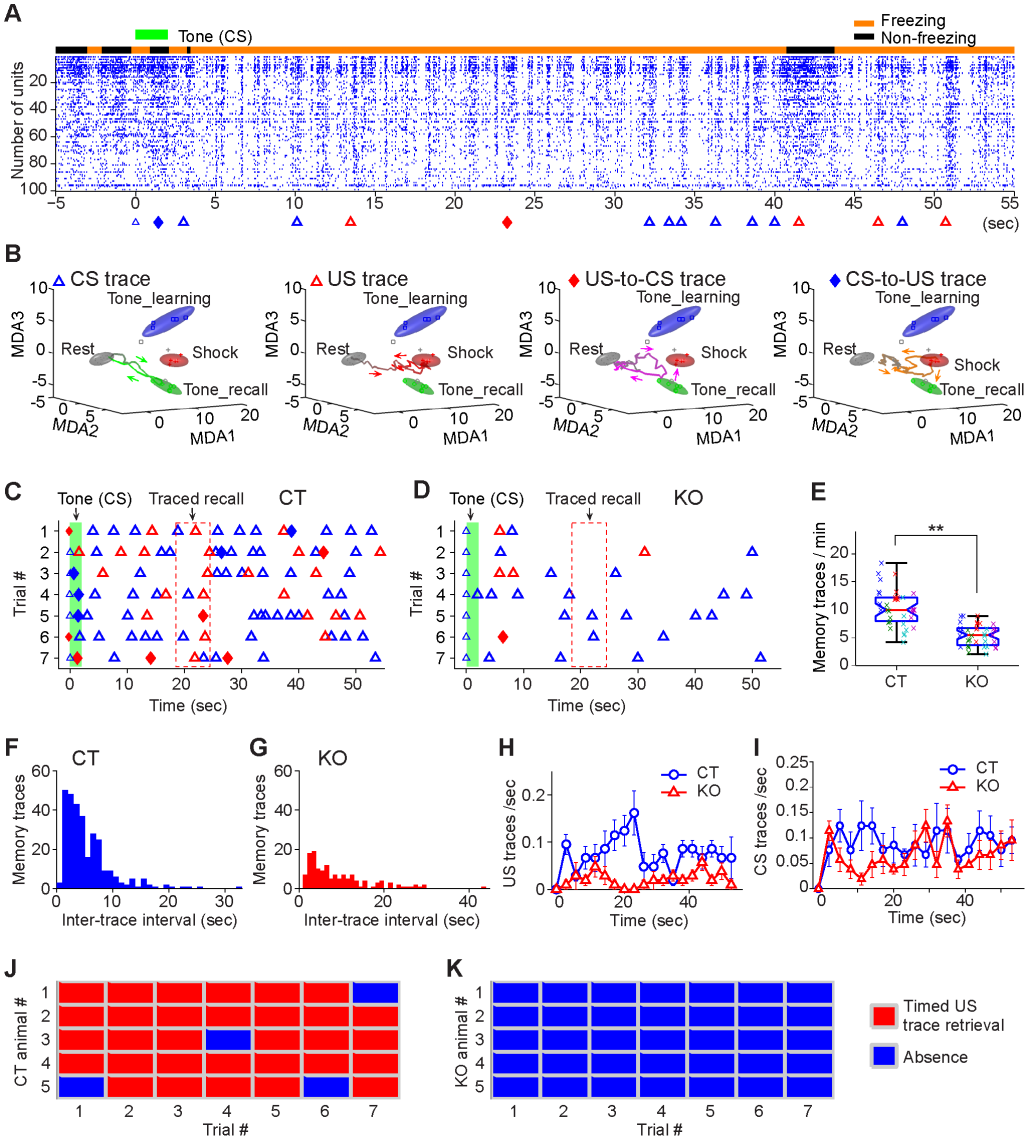
Real-time fear memory traces during the trace retention test. (**A**) An example of various memory traces being retrieved during the 60-sec traced fear recall test in a control mouse (1-hr retention). A conditioned tone (2-sec) was presented without the reinforcing foot shock. The black bar on the top of spike raster indicates the non-freezing state, whereas the orange bar indicates the freezing state of the animal. Colored triangles or diamonds at the bottom indicate the various moments at which those memory traces were retrieved. (**B**) Examples of each type of memory traces retrieved during the traced recall. (**C**) Various memory traces being retrieved over all seven tone trials from a representative control mouse. Symbols: simple CS trace, blue triangle; simple US trace, red triangle; US-to-CS associative trace, red diamond; CS-to-US associative trace, blue diamond. The time window for recalling the anticipated foot shock memory is illustrated by dotted rectangle (18.5-24.5 seconds after the termination of the conditioned tone. Please note the simple or associative foot-shock traces at this time period. (**D**) Greatly reduced numbers of memory traces retrieved in the knockout mice during 1-hr traced retention test. Please note the lack of the simple US or associative US-to-CS traces at this trace-interval time period. (**E**) The average memory trace retrieval rates in the control and knockout group during 1-hr trace recall test (the central red lines are the medians, the edges of the box are the 25th and 75th percentiles, each row of ‘x’ markers indicate the result of seven trials from one animal, small random numbers were added to avoid overlap of the markers; student *t*-test, **p<0.01). (**F**) Memory trace-time interval analysis revealed that trace recall in the control group has the characteristics of exponential decay distribution, indicating it occurred in a ‘bursting’ manner. (**G**) Memory trace retrieved in the mutant mice did not show tight an exponential decay process. The inter-trace intervals of control and mutant mice formed different distribution (Two-sample Kolmogorov-Smirnov test, *p = *5.3E-7). (**H**) Time distribution of US traces (include US simple trace and US-to-CS trace) in the control mice showed a distinct peak at about 22 second time-window at which a foot shock would be anticipated. This peak was absent in the mutant group. One way ANOVA test between two curve, *p = *1.9E-6. (**I**) Time distribution of CS traces did not reveal any significant peaks in either control mice or knockout mice. (**J**) The occurrence of real-time shock memory traces around the 20-sec traced interval time window (18.5–24.5 sec after the offset of the recall tone) for all seven trials in five control mice. Red rectangles represent the occurrences of the correct foot-shock memory traces, whereas blue squares indicate the absence of foot-shock memory traces at the time point. (**K**) Lack of memory traces for time relationship in predicting foot shock event and its timing in five knockout mice. There is a significant difference in recalling memory of time relationship between CS and US between genotypes. Wilcoxon rank sum test, *p<*0.01.

Formation of trace fear conditioning memories offers a unique paradigm for assessing both emotional fearful memory and memory of timing relationship on “what and when” information [34]–[36], [42]. To qualify any ensemble traces observed during learning as memory traces, these traces must re-appear upon cue-induced memory recall. Therefore, we subjected the mice to trace retention test by placing the animals to a non-conditioned chamber different from the original conditioning chamber. 2-sec tone was played in the absence of foot shock, and it was then repeated for seven times with the 1–3 minutes randomized interval between each tone representation. Prior to the exposure to the recall tone, the mice had a low amount of freezing (Figure 10A, see the top black bar). Shortly after hearing the conditioned tone, the control animals exhibited significantly more freezing (Figure 10A, see the orange bar). Our MDA/sliding window decoding analysis revealed that the conditioned tone led to a string of CA1 memory traces re-appeared in the trace recall session (Figure 10A, see triangles and diamonds at the bottom of the raster plot). In the control mice these transient dynamic trajectories included simple CS traces and US traces, as well as US-to-CS associative memory trace and CS-to-US associative traces (Figure 10B). On average, various memory traces were retrieved with a rate up to 17 memory traces per min in the controls (1-min recall session) (Figure 10C).

By contrast, the numbers of memory traces retrieved from the knockout mice were much lower, and few occurred in form of associative trajectories (Figure 10D). On average, the control mice retrieved 10.4 memory traces per recall, verses 5.5 memory traces per trial in the mutant mice (Figure 10E). Interestingly, the memory trace interval analysis revealed that memory retrieval followed the exponential decay-like process (Figure 10F). This exponential decay distribution reflected that retrievals of memory traces were formatted closely together in time, with two or more traces temporally clustered together (see Figure 10C). The shortest time interval between two individual memory traces was 0.76 sec. In comparison, the retrieved memory traces from mutant mice were significantly degraded in term of its bursting temporal format (Figure 10G). A two-sample Kolmogorov-Smirnov test indicated that the inter-trace intervals of control and mutant mice had different distribution (*p<*0.001).

One unique property of Pavlovian trace conditioning is that it offers the opportunity to examine whether and how the brain generates real-time memory traces of “what at when” time information regarding the temporal relationship between hearing the tone and predicting the timing at which US event should follow [36], [42]. By plotting the time distribution of various real-time memory traces retrieved over the trace retention test period, we found that the US memory traces exhibited a distinct recall peak in the control mice, centered around 20 seconds after the offset of the 2-sec tone (Figure 10H, blue line). This showed that the hippocampus in the control mice consistently predicted the arrival time of foot-shock at that particular moment. The mutant mice failed to show the memory trace of “what at when” time relationship (Figure 10H, red line).

On the other hand, the time distribution plot for CS traces over the 1 min recall period in the control mice did not reveal any significant peak, suggesting that this is a specific phenomenon in term of causal relationship between CS hearing the tone and US memory trace retrieved with 20 seconds of time interval (Figure 10I).

As a group, all five control mice showed consistent retrievals of the US ensemble traces around the 20-sec time interval (Figure 10J, red rectangles). In contrast, all of the knockout mice failed in predicting US events at this moment in time (Figure 10K). Therefore, our results demonstrated real-time memory traces encoding “what event at when” are critically dependent on the NMDA receptors.

### Real-time hippocampal memory traces during contextual recall test

**Figure 11:**
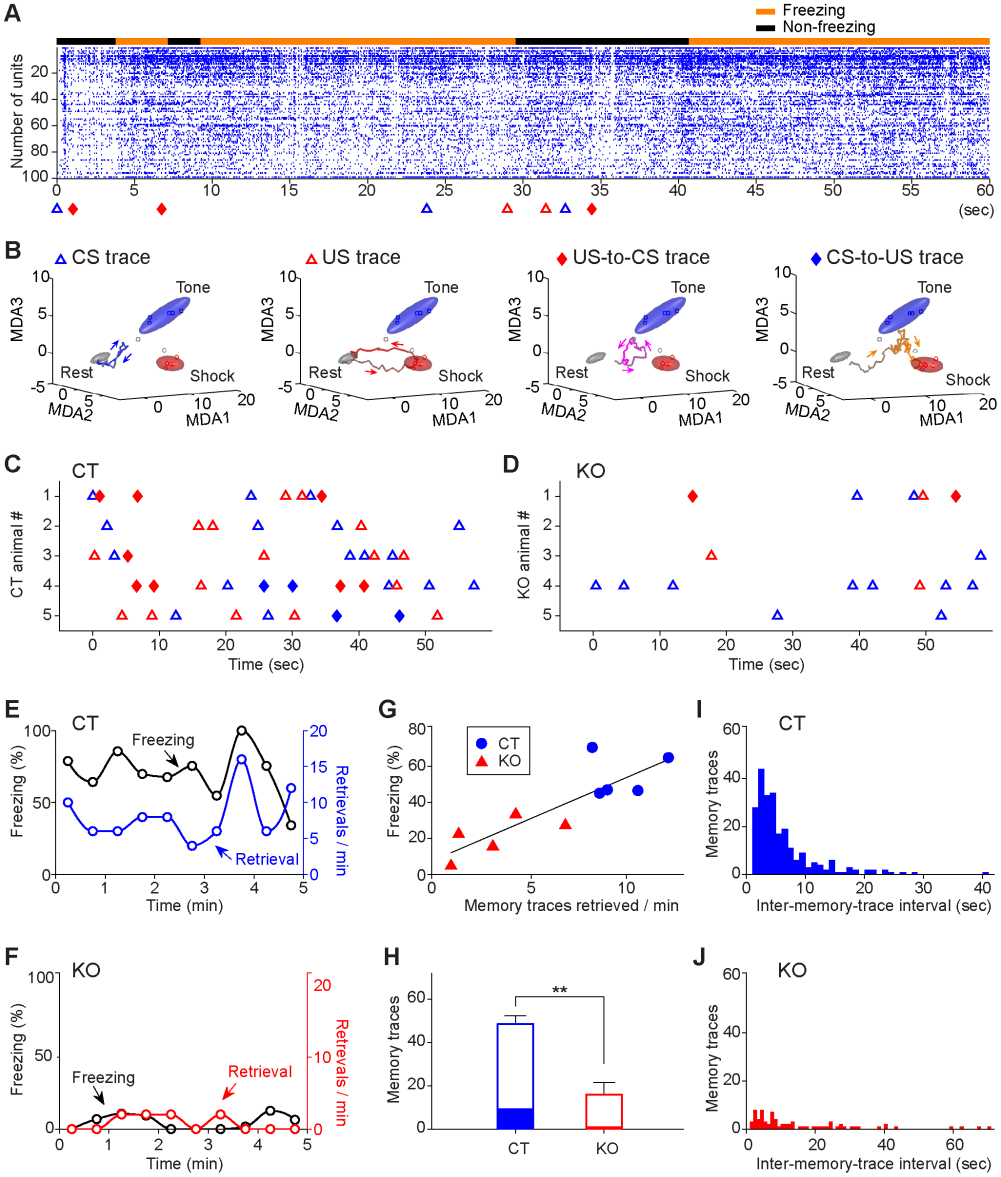
Real-time fear memory traces during the contextual retention test. (**A**) An example of various memory traces being retrieved during the 1-hr contextual fear recall test (first 60 sec shown here). The black bar on the top of spike raster illustrates the non-freezing state, whereas the orange bar indicates the freezing state of the animal. Note that the two initial memory traces were recalled ∼3–4 seconds before freezing behavior once the animal returned to the conditioning chamber. Colored triangles or diamonds at the bottom the raster indicate the moments at which those memory traces were retrieved. Memory traces were detected in both freezing and non-freezing states. (**B**) Examples of four types of memory traces retrieved during the contextual recall. (**C**) Memory traces retrieved over the first 60-sec of contextual retention tests in all five control mice. Symbols: simple CS trace, blue triangle; simple US trace, red triangle; US-to-CS associative trace, red diamond; CS-to-US associative trace, blue diamond. (**D**) Reduced numbers of memory traces during 1-hr contextual recall test in the five knockout mice. (**E**) Freezing responses correlated with memory trace retrievals in a control mouse in the 5-min contextual retention test. (**F**) Lower freezing and lower numbers of retrieved memory traces in a mutant mouse in the 5-min contextual test. (**G**) Linear regression analysis shows that at group level, averaged freezing responses also correlated with their averaged numbers of total pattern retrievals (*r^2^* = 0.73, *p<*0.01). Each blue dot represents the data from a single control mouse and each red triangle represents the data from a single knockout mouse. (**H**) The total numbers of memory traces retrieved in the control and knockout mice (Wilcoxon rank sum test, **p<0.01; error bars represent SEM). The filled bar portion represents the associative memory traces retrieved during the contextual retention test. The knockout mice had few associative memory traces retrieved (Wilcoxon rank sum test, *p<*0.05). (**I**) Inter-memory trace-time interval analysis revealed that contextual recall in the control mice has the characteristics of exponential decay distribution. (**J**) Memory trace retrieved in the mutant mice did not show obvious exponential decay process temporal associativeness. There is significant difference between memory trace time distribution from control and mutant mice (two-sample Kolmogorov-Smirnov test, *p = *1.2E-7).

Trace fear conditioning also offered us an opportunity to examine contextual fear memory in the CA1 [8], [43]–[46]. However, the animals can recall contextual fear memory at various time points, the traditional peri-event spike histogram analysis could not be readily performed due to apparent lack of the time zero for the averaging spike raster. We took advantage of MDA/sliding window decoding technique and mapped the retrievals of fear memory traces during the entire contextual retention test period. We found that immediately after the control mouse entered the conditioning chamber (in the absence of the tone), fear memory traces were retrieved within a few seconds (see examples in Figure 11A). The re-emergence of fear memory traces preceded the freezing behavior (see the orange bar above the raster and triangle and diamond below the raster in Figure 11A). Similar to those observed in trace retention tests, the retrieved memory traces during contextual recall included various simple and associative memory traces (Figure 11B). In all five control mice, we have observed the recalling of various memory traces (See the first 60 sec period after the mice entered contextual retention chamber in Figure 11C). In contrast, the retrievals of CS or US-related traces in the knockout mice were significantly reduced during the contextual retention tests (Figure 11D).

Interestingly, the number of memory traces retrieved during contextual recall is correlated with the amount of contextual freezing within individual control animals (Figure 11E). The mutant mice had lower amount of freezing and reduced numbers of memory trace retrieved (Figure 11F). Linear regression analysis of the group data further showed the correlation between the numbers of memory traces retrieved and freezing responses (Figure 11G, *r^2^* = 0.73, *p<*0.01), with control mice at the higher end of the freezing and memory trace recalled and mutants at the lower end of the plot. Calculation of total memory traces retrieved during the entire 5-min contextual recall showed that control mice recalled at 48.6±3.7 traces, whereas the mutant mice had only 16.0±5.4 traces (Figure 11H). We noted that about 20 percent of memory traces retrieved in the control mice were in forms of associative memory traces during contextual retention tests (Figure 11H, solid portion of the bar), but very few was in the mutant mice. This is consistent with impaired formation of associative memories observed in learning phase in these mutant mice.

To further quantify the temporal effects of NMDA receptors on retrieval dynamics of contextual memory recall, we plotted the inter-memory trace-intervals among all memory traces retrieved during this 5-min retention test period. The control mice recalled fear memories, often with two or more memory traces tightly clustered together (Figure 11I). The shortest inter-memory time interval was at 1.02 sec. However, the mutant mice lacked exponential decay-like distribution and were significantly impaired in temporal dynamics (Figure 11J, two-sample Kolmogorov-Smirnov test, *p = *1.2E-7).

### Temporal map of CA1 fear memory codes’ contents and associational dynamics

**Figure 12:**
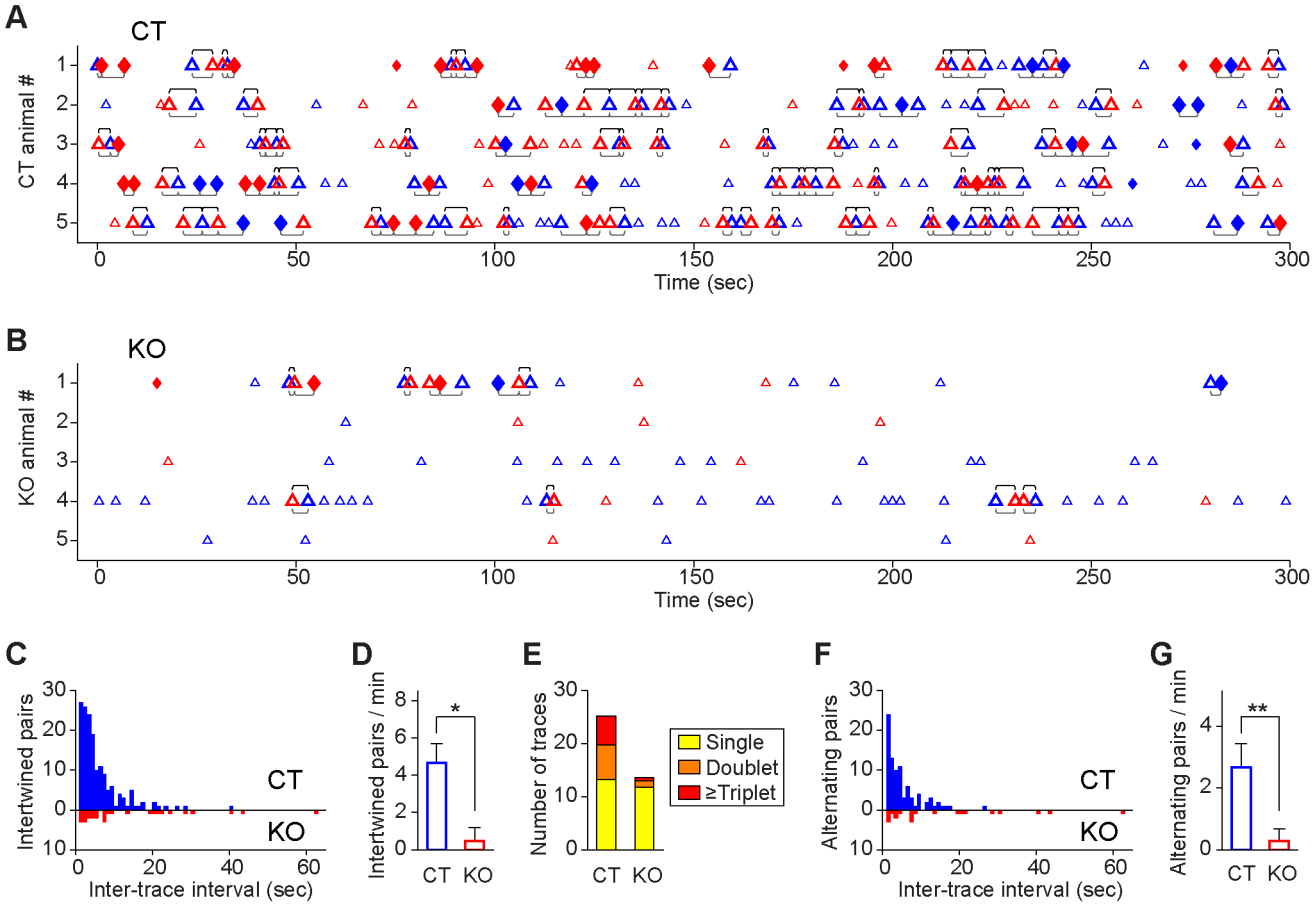
Temporal map of CA1 fear memory neural codes underlying contextual memory recall. (**A**) The intertwined memory trace retrievals (defined as retrievals between individually distinct memory traces, see underlined brackets) and alternating retrieval dynamics (defined as retrievals between distinct simple traces, upper brackets) were prevalent in all five control mice during the 5-min contextual recall. Symbols: CS trace, blue triangle; US trace, red triangle; US-to-CS associative trace, red diamond; CS-to-US associative trace, blue diamond. The intertwined retrievals between different traces within 7 sec time window are underlined, whereas alternating retrievals between US and CS or CS and US are marked by upper brackets. (**B**) Degraded memory codes in five mutant mice during 5-min contextual retention tests. Only KO #1 had five associative traces during the recall, all other mutants failed to produce such traces. Intertwined and alternating retrieval patterns were greatly diminished. (**C**) The intertwined memory retrieval in the control group (blue plots) during contextual recall follows an exponential decay process, indicating that intertwined memory traces were recalled in temporal clusters. The mutant mice were significantly impaired in the temporal association between recalled memory traces (Red plots) (two-sample Kolmogorov-Smirnov test, *p = *0.014). (**D**) Significant differences in the average occurrences of intertwined memory trace pairs between the control and knockout mice (Wilcoxon rank sum test, **p<0.01; error bars represent SD). (**E**) Numbers of various temporal structures of memory traces in single, doublet, triplet across the control and mutant mice during contextual recall. (**F**) The alternating retrieval for US simple trace followed with a simple CS memory trace or CS simple trace followed by a US memory trace in the control mice also exhibited exponential decay distribution, but not in the mutant mice (two-sample Kolmogorov-Smirnov test, *p = *0.0042). Trace interval for 50% temporally associational recall in the control is 3.6 sec for control mice and 8.2 sec for mutant mice. (**G**) Differences in the alternating retrieval rates between the control and knockout mice (Wilcoxon rank sum test, **p<0.01; error bars represent SD). To appreciate temporal associativeness of memory recalled, memory patterns from a control (Mouse #4) and mutant mouse (Mouse #3) were converted into audio clips of “Pavlovian memory symphony” (Sound S1 and S2 correspondingly).

To seek out additional understanding of the temporal organizing patterns in relation with dynamics of associative memories, we analyzed the various relationships among and between complex associative memory traces and simple memory traces during both contextual and traced recall. We first examined how many associative memory traces (i.e. CS-to-US or US-to-CS associative memory traces) were retrieved during the entire 5-minute contextual recall session. We found that the control group retrieved a total of 47 such associative memory traces recalled (shown as red or blue diamonds) (Figure 12A). Most interestingly, these associative memory traces often retrieved in alternating doublets or more. We referred the alternating retrieval pattern when a given associative memory trace was followed by another associative memory trace. On the other hand, when a given associative memory trace was followed by other simple traces, we termed it as intertwined retrieval pattern (Figure 12A). By comparison, the mutant group had a much lower number, with only 5 associative memory trace-like patterns detected (all came from one knockout mouse) during this 5-min contextual retention test (Figure 12B).

Next we examined the temporal associational relationships among simple CS traces and US memory traces retrieved during contextual recall. We found that many CS or US traces were retrieved in a temporally associational manner as evident from the three following analyses: 1) *the intertwined retrieval pattern*, again, it referred to the temporal format when a given CS or US simple memory trace was followed by a different memory trace (either a simple or associative memory trace). 2) US trace-based *alternating retrieval pattern* (when a simple US memory trace was followed by a simple CS memory trace); 3) CS trace-based *alternating retrieval pattern* (when a given CS trace was followed by a US memory trace).

In the control mice, the vast majority of memory traces retrieved occurred not only closely in temporal domain (in clusters), but also using the intertwined format (Figure 12A). These intertwined pairs (underlined) also exhibited exponential decay (Figure 12C). In comparison, memory retrieval in the mutant mice had a loose temporal format, with few doublets or triplets (Figure 12B,E). For those simple traces retrieved in the mutant CA1, they showed little intertwined retrieval patterns and lacked exponential decay distribution (Figure 12C, see the red histogram). There was a significant difference in the total pairs of intertwined memory recall between the genotypes (Figure 12D, Student’s *t* test *p*<0.05). In the control, about half of the all memory traces retrieved occurred as clusters in doublet or triplet formats, whereas the mutant mice generally lacked doublet or triplets (Figure 12E).

Similarly, in control mice, the vast majority of the simple US traces followed by simple CS memory traces or vice versa (alternating retrieval pattern) within a 6.6 sec time window (Figure 12F, blue histogram). Again, this temporally alternating recall patterns were impaired in the mutant mice (Figure 12F, red histogram. Two-sample Kolmogorov-Smirnov test, *p = *0.0042). The average occurrence in CS/US alternating recall formats within the interval less than 7 sec in the control mice was 2.7 times per minute, whereas the knockout mice had only about 0.3 time per minute (Figure 12G, Student’s *t* test *p*<0.01).

**Figure 13:**
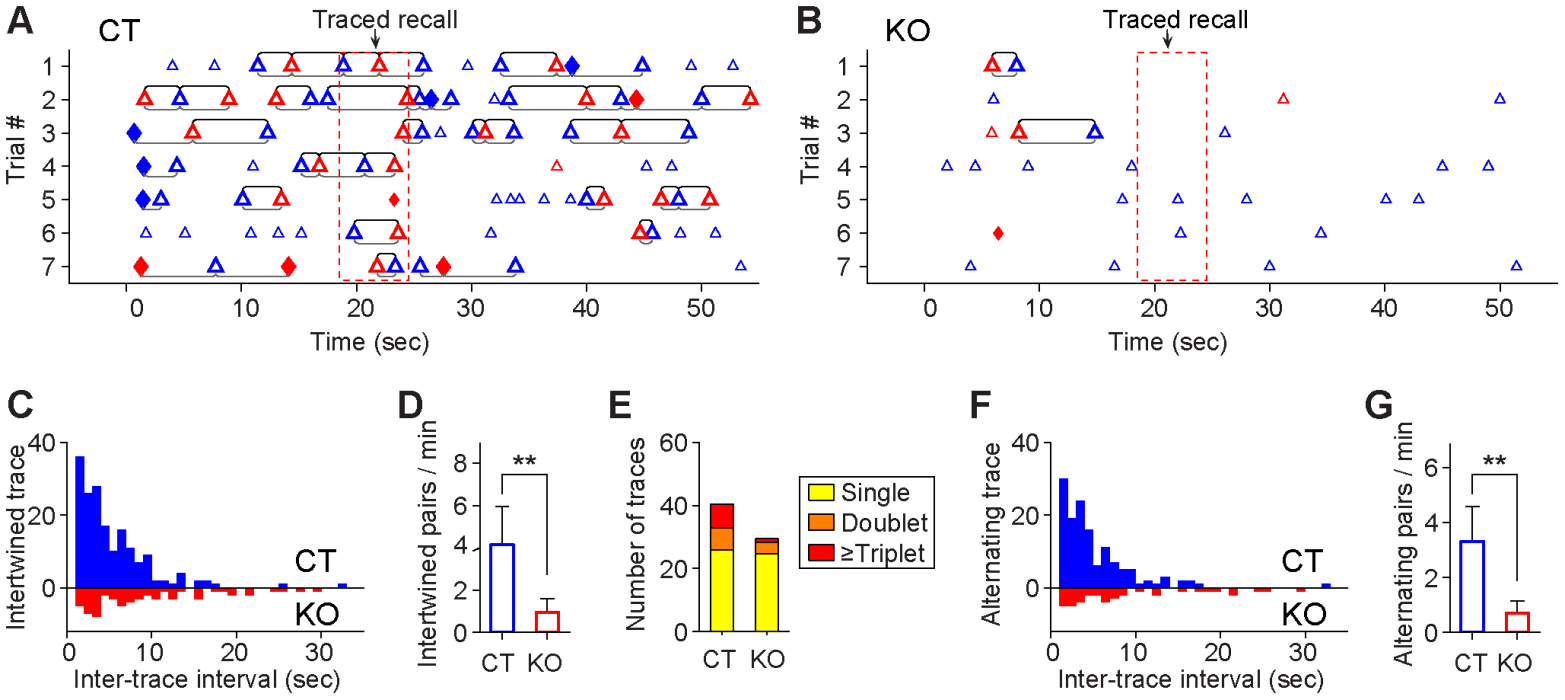
Temporal map of CA1 fear memory neural codes during tone-traced retention test. (**A**) The intertwined memory trace retrievals (underlined brackets) and alternating retrieval (upper brackets) map in a control mouse over seven tone-traced recall trials. Note the timed retrieval of foot shock memory traces around the 22-second time point after the onset of the conditioned tone. (**B**) Lack of temporally intertwined and alternating recall structures in memory codes of the knockout CA1 region during the tone-traced recall trials. (**C**) The intertwined memory retrieval in the control group during tone-traced recall exhibited clear exponential decay distribution, but not in the knockout mice (two-sample Kolmogorov-Smirnov test, *p = *0.0024). Time window for 50% temporally associational recall is 4.1 sec for control mice and 7.1 sec for mutant mice. (**D**) Significant reduction in intertwined memory retrievals during tone-traced recall in knockout mice as compared to the control group (Wilcoxon rank sum test, *p<0.05; error bars represent SD). (**E**) Average number of temporal structures of memory traces as single traces, doublet, triplet the control and mutant mice during trace recall. (**F**) The alternating retrieval between simple US trace and simple CS memory trace in the control mice exhibited an exponential decay distribution, suggesting that the retrieval occurred as clusters in time domain. Such temporal association is nearly absent in the mutant mice (two-sample Kolmogorov-Smirnov test, *p = *0.022). Trace interval for 50% temporally associational recall in the control is 3.8 sec for control mice and 6.6 sec for mutant mice. (**G**) Differences in alternating retrieval rates during tone-traced recall between the control and knockout mice (Wilcoxon rank sum test, *p<0.05; error bars represent SD).

To investigate whether the intertwined and alternating recall dynamics represent a general temporal mechanism for ensuring associative memory recall associational in its time domain, we further analyzed the traced fear recall in both control and knockout mice (Figure 13A,B). Again, we found that CS-to-US and US-to-CS associative traces were mostly observed in the control mice (Figure 13A), but rarely in the mutant mice (Figure 13B). When using 7 sec as the time threshold, about 36% of various memory traces were dynamically retrieved in doublet, triplet or more in the control mice, whereas the mutant mice had little such bursting manner (Figure 13E). More importantly, these bursting memory retrieval patterns occurred in intertwined formats (underline) in the control mice as confirmed by the exponential distribution of intertwined recalling pairs (Figure 13C, blue histogram). The mutant mice failed to exhibit any significant intertwined dynamics (Figure 13C, red histogram). The total numbers of the intertwined recalling occurrences in the control was at 4.1 times per min, vs. 0.9 times per min in knockout mice (Figure 13D, Student’s *t* test *p*<0.01).

Finally, our analysis also shows that alternating retrieval patterns (upper line) between CS/US traces were prominently present in the normal hippocampus, as evident from the exponential distribution in the control mice (Figure 13F). About 75% of alternating traces in which US memory traces were followed closely by a CS memory trace or vice versa, within a time window of 6.7 sec in the control mice, whereas the mutant mice had very limited such recalling patterns (Figure 13F). The total number of alternating recalls in the control mice was at 3.3 times per min, whereas the number for the mutant mice was at 0.7 times per min (Figure 13G, Student’s *t* test *p*<0.01). Taken together, these results showed that retrievals of Pavlovian fear conditioning memories in the control mice were highly associational in its time domain, with many memory traces organized in the intertwined or alternating recalling formats. Inducible NMDA receptor knockout disrupted such temporal organization of memory traces in the CA1.

## Discussion

Mapping brain activity patterns and then understanding their underlying meaning are an emerging interest among many brain researchers [10]–[13]. By employing large-scale neural recording techniques and pattern classification mathematical tools, we systematically mapped and decoded real-time CA1 fear memory traces during learning as well as trace and contextual recall. The initial successful decoding of real-time fear memory’s neural code was first reported in the wild-type mouse hippocampus [13], [36]. Here, we have further examined the hippocampal fear memory engrams in inducible and forebrain-specific NMDA receptor knockout mice. From our present analyses of large datasets using dimensionality-reduction pattern classification techniques, we have made follows sets of novel observations that have not been reported previously:

First, CS or US stimulation can still generate significant CA1 ensemble representation in the knockout mice, suggesting that a good level of perceptual real-time patterns was produced with discriminative separation even in the absence of the NMDA receptor in the forebrain. Yet, while the hippocampus of control mice readily generated US-to-CS or CS-to-US associative memory traces upon repeated pairing of CS and US stimuli, the mutant mice failed to do so. To our knowledge, this represents the first network-level evidence for the NMDA receptor’s role in the formation of *real-time* associative fear memory traces in the hippocampus. It is interesting to note that CS-paired foot-shock produced a smaller set of units in the mutant mice. This may suggest several possibilities including the reduced perceptual representation of foot shock in the mutant CA1. However, given the highly convergent inputs from the multi-modal cortex to the hippocampus, these reduced CS-paired foot shock responsive cells in the mutant CA1 region may reflect the loss of associative binding or integration with contextual or environmental information with US stimuli. It might be of interest to examine the memory traces by using unpaired CS and US protocols in future. Some of the foot-shock memories most likely contain the context or location information that integrates “what and where” memory traces. Due to the smaller size of the mouse fear conditioning chamber and high proportion of freezing during learning and recall, place cell property, which usually requires running behavior for characterizations, has prevented us to study in the current protocol. Additional experimental designs [47] will help address this issue in future.

Second, various CA1 ensemble traces underwent on-line reverberation during the training in the control mice. Interestingly, the mutant mice had greatly reduced reverberation. Recent studies have reported that trace fear conditioning caused changes in membrane potentials [48] or synaptic potentiation in the hippocampus and prefrontal cortex [49], [50]. The potentiated excitability in neurons or their synapses may contribute to the initiation of pattern reverberations in the network. The strong correlation between on-line memory trace reverberation and immediate freezing supports the idea that both phenomena reflect learning process, with one at neural population level, other at behavioral level. Diminished memory trace reverberation explains well why blockade of NMDA receptor pathway would impair fear memory formation or consolidation [22], [31], [51]–[53]. By restricting inducible knockout of the NMDA receptor to the learning or post-learning consolidation window, the distinct temporal roles of the NMDA receptor can be further studied [54]–[56].

Third, our study has provided the activity map on real-time CA1 fear memory contents and their temporal dynamics. We showed that memory retrievals conformed to a set of dynamic patterns or templates. Overall, fear memory traces were retrieved in clustered or burst templates in the CA1 of control mice, at a frequency range of 7.2 to 13.9 times/min during contextual or tone recall. In contrast, the inducible knockout mice had much lower memory content, limiting to the 0.8 to 7.0 traces/min. Its poor memory content is further evident from the near absence of US-to-CS or CS-to-US associative memory traces during contextual or tone recall. This suggests the qualitative differences in holistic memory representation between the mutant mice and their control littermates.

Fourth, we have uncovered two important temporal dynamics in organizing associative memory patterns in the hippocampus: 1) Many distinct memory traces are retrieved in a highly intertwined manner, and invariantly in a memory trace bursting format (as shown by an *exponential decay* process); 2) Most of simple memory traces in the control mice were also retrieved in an alternating fashion (i.e. retrieving a US trace and then followed by a CS trace, or vice versa within the 7 sec time-domain, also an exponential process). Such intertwined and alternating retrieval dynamics are consistently observed during both the contextual and tone recall in the control mice. This recalling dynamics may serve as a key temporal organizing mechanism in generating the holistic memory engrams *associational in its time domain.* It will be of great interest to examine how various manipulations of experimental conditions, such as pre-context exposure or reconsolidation protocols, change fear memory engram [57]–[59].

Finally, the intertwined and the alternating retrieving dynamics are almost completely missing in the inducible NMDA receptor knockout mice. Despite CS or US traces could still be detected the mutant CA1 region, their retrieval did not conform to the temporal association templates. The lack of memory of time for predicting the 20-sec CS-US trace interval in the mutant mice provides the first direct evidence that the NMDA receptor-mediated plasticity is essential for producing “what at when” time memory traces that have been described in the hippocampus [21], [36]. The working memory for tracking trace fear memory has been reported to involve cholinergic regulation in the hippocampus and other cortical areas [60], [61]. Another neuromodulatory candidate mechanism might be dopamine [62]–[64]. This memory of time is likely an emergent result of the hippocampus interactions with the VTA, anterior cingulate cortex, amygdala, perirhinal cortex, and other higher cortical regions [21], [64]–[69].

In summary, by decoding CA1 activity patterns underlying Pavlovian fear conditioning, we have uncovered the real-time neural codes and temporal organizing patterns of trace and contextual fear memory engrams in the presence or absence of the NMDA receptors. Our study has, for the first time, described precisely the multiple actions of the NMDA receptors on regulating dynamic information contents and temporal patterns of real-time fear memory engrams in the brain.

## Materials and Methods

### Ethics statement

All procedures were conducted in accordance with the National Institutes of Health guidelines and with the approval of the Committee on Animal Care at the Georgia Regents University (GRU) and Banna Biomedical Research Institute (BBRI).

### Production of inducible and region-specific NMDA receptor knockout mice

The inducible NMDA receptor knockout (KO) mice were generated as described previously and maintained on BCF hybrid background (B6 x CBAF1) [7] _ENREF_1. The KO mice are homozygous for the floxed-*NR1* gene and heterozygous for the CaMKII-*Cre* transgene, the *NR1-GFP* transgene under control of the tet-O promoter, and the tetracycline transactivator (*tTA*) transgene, which is driven by the β-actin promoter and contains a floxed stop sequence (*fNR1*/*fNR1, Cre*/+, *tTA*/+, and *NR1-GFP*/+). The littermates lacking the Cre gene (*fNR1*/*fNR1, tTA*/+, *NR1-GFP*/+; or *fNR1*/+, *tTA*/+, *NR1-GFP*/+) were used as control mice. For genotyping, southern blot method was used to detect the floxed NR1 (*fNR1*) gene and the protocol is the same as described [70]. About 10 µg-purified tail DNA were digested by EcoR I, fractionated by electrophoresis on 0.7% agarose gels and transferred onto Zeta-probe GT membranes (BioRad). A 1.2 kb DNA fragment of 3′ NR1 gene probe was labeled by α-32P-dCTP and hybridized to the GT membranes. PCR detection of the *Cre, tTA*, and *NR1-GFP* transgenes, approximately 0.5 to1 µg of mouse tail DNA was amplified in PT100 thermal cycler using the programs as follows: 1 minute, 94°C; 45 sec, 55°C; and 1 min, 55°C for 35 cycles. The primers for *Cre* detection is 5′-AGA TGT TCG CGA TTA TC and 5′- AGC TAC ACC AGA GAC GG; for *tTA* detection is 5′- CAA TTA CGG GTC TAC CAT and 5′-GGT TCC TTC ACA AAG ATC CTC; and for *NR1-GFP* detection is 5′-GGT AGA GCA GAG CCC GAC CCT and 5′-GTA TCT GGA AAA GCA CTG respectively. The size of specific PCR products for *Cre* is 490bp, 450bp for *tTA*, and *NR1-GFP* 400bp for NR1-GFP. The primers for floxed NR1 is fNR1-1: 5′-GTGAGCTGCACTTCCAGAAG; fNR1-2: 5′-GACTTTCGGCATGTGAAATG; fNR1-3: 5′-CTTGGGTGGAGAGGCTATTC; fNR1-4: 5′-AGGTGAGATGACAGGAGATC. The PCR between fNR1-1 and fNR1-2 is ∼160bp band for detecting the wild type NR1, and ∼280bp band between fNR1-3 and fNR1-4 for detecting the fNR1 allele.

In our experiments, the inducible switch-off of the NMDA receptor function occurred 5 days before our recording experiments when we fed the inducible knockout mice with food pellets containing dox at 6 mg/g. This feeding protocol has been shown to disable NMDA receptor function within 3∼5 days in the hippocampus and cortex in freely behaving mice [7]. Our previous study showed that the cre/loxP-mediated deletion occurred in about 97% of CA1 pyramidal cells and the averaged 60% of cortical principal neurons (CaMKII promoter active neurons). We maintained these mice on dox food throughout the experiments until the mice were sacrificed for histological verification of electrodes position.

### *In vivo* recording and spike sorting

128-channel recording arrays were constructed as previously described in details [39] and used to record neural activity from hippocampal CA1 region in freely behaving mice (5-6 month old, males) [36]–[40]. Each mouse was implanted with two independently movable bundles of 32 steretrodes (64 channels on each side of the hippocampi) to bilateral hippocampi under deep anesthesia using 60 mg/kg ketamine (Bedford Laboratories, OH) and 4 mg/kg Dormitor (Pfizer Animal Health, NY). The electrode bundles were positioned above the dorsal hippocampi (2.0 mm lateral to bregma and 2.3 posterior to bregma on both right and left sides). After the mice recovered from surgery, the electrodes were advanced slowly, over next five to ten days, in daily increments of about 0.07 mm until the tips of the electrodes reached the pyramidal layers of the hippocampal CA1 region.

The spike activity was recorded using Plexon Systems (Dallas) and then sorted using the MClust 3.3 program (http://redishlab.neuroscience.umn.edu/MClust/MClust.html). First, the recorded data were filed as Plexon system format (*.plx). Before spike sorting, the artifact waveforms were removed and the spike waveform minima were aligned using the Offline Sorter 2.8 software (http://www.plexon.com, Dallas, TX). The aligned data were then saved as files in Neuralynx system format (*.nst). After that, the MClust 3.3 program was used to isolate different spiking units. Only units with less than 0.5% of spike intervals within a 1 msec refractory period and with clear boundaries, judged by L-ratio and Isolation distance calculation [71], were included in the present analysis (S. Fig. 1). Identification of different responsive and nonresponsive units from the same electrodes to tone and foot shocks are consistent with the notion that artifacts from external stimuli were removed and did not contaminate the recording results. Moreover, the observation that responsive units responding to foot-shock usually changed firing exceeding the stimulus duration (i.e. foot shock lasted only for 285-msec) further ruled out the contamination of electrical noise or artifacts.

Ten sets of recording data were obtained from five control and five mutant mice for the current analysis. Isolated units were identified. Further analysis is based on the sorted data; therefore, no electric artifacts were included during the shock events. To confirm the recording sites of the electrodes, 10 µA current was applied to each recording electrode for 5 seconds in order to mark the positions of the stereotrode bundles. Nissl staining confirmed the electrode positions. The stability of the ensemble recordings were judged by comparing waveforms and interspike intervals at the beginning, during, and after the experiments.

Only stably recorded units throughout the experiments are included in the present analysis. Recorded units were classified into putative pyramidal cells and fast-spiking putative interneurons. Putative pyramidal cells were defined as units with relatively wide waveforms (trough-to-peak length ≥300 µs) and lower average firing rates (<5 Hz), whereas putative interneurons were identified as units had relatively narrow waveforms (<250 µs) and higher firing rates (>5 Hz) [37]. Pyramidal cells are known to fire complex-spike bursts. To measure the characteristic burst activity, the proportion of inter-spike intervals that are shorter than 10 msec among all the inter-spike intervals was examined as the burst index.

By calculating waveforms widths and firing rates putative pyramidal cells and fast-spiking putative interneurons, we found that units’ overall basic properties were largely similar between the control and knockout animals. Overall firing rate of putative pyramidal cells were about 1.46±0.05 Hz during sleep state and 1.73±0.06 Hz during awake in the control group, whereas the firing rates for putative pyramidal cells from the KO mice were 1.51±0.05 Hz during sleep and 2.03±0.08 Hz during awake. The spike widths of putative pyramidal units were 446.47±3.58 in CT mice and were 441.49±3.76 in KO mice. The burst index for the putative pyramidal cells was 13.48±0.44% for the control and 10.14±0.41% in KO mice during sleep (Student’s *t* test *p*<0.001). During wakeful states, burst index was 13.12±0.41% for the control and 10.70±0.42% for the mutant mice (Student’s *t* test *p*<0.001), suggesting a slight decrease in bursting upon the forebrain excitatory neuron-specific NMDA receptor knockout. On the other hand, there is no difference in the overall firing rate or waveform width of fast-spiking putative interneurons between the genotypes. The fast-spiking units from the control mice were about 13.44±0.85 Hz during sleep and were 14.92±0.92 Hz during awake, whereas the fast spiking units from the KO mice were at 13.65±0.82 Hz during sleep and 16.06±1.03 Hz during awake. The spike widths of putative interneurons were 194.39±3.98 in CT mice and were 185.00±3.97 in KO mice. This is consistent with the fact that the deletion of the NMDA receptor was restricted to excitatory neurons under the control of the CaMKII promoter [6], [7].

### Fear conditioning task

The fear conditioning chamber was a square chamber (10" × 10" × 15") with a 24-bar shock grid floor, and the trace recall chamber was a distinct semicircular shape chamber with a smooth and opaque floor. Freezing responses of animals in the chamber could be observed by experimenters and were videotaped [5]. Before training, the mouse was habituated in both chambers for five minutes per day, and three days in total. When the mouse was habituated in contextual chamber a tone was played 10 times. This protocol facilitates the formation contextual memory as well as trace fear memory in rodents [36], [57].

On the training day, the recording began with a 1-h pre-training sleep period in the home cage (a plastic tub where the mouse lived) and then followed by a 3-min pre-training exploration period in the shock chamber and then a 30-min pre-training period during which a 2-sec tone (85 dB continuous tonic sound at 2.8 kHz) was played ten times at random intervals. This allowed us to profile the CA1 responses to naïve tone. The animal was then brought back to the home cage for a 30-min break before trace fear conditioning began. During trace conditioning, the conditioned stimulus (tone, 2-sec, 85 dB continuous tonic sound at 2.8 kHz) was used, but now as conditioned stimulus (CS) paired the unconditioned stimulus (a continuous 285-msec foot shock at 0.75 mA) with a 20-sec interval (after the off-set of the tone). This CS-US pairing was repeated for seven times, with 1-3 min random time intervals between each pairing. The mouse was then brought back to the home cage for 1-h post-training rest. The immediate freezing was calculated from the first 30 seconds after each shock.

After a one-hour rest in the home cages, memory retention/recall tests in the contextual and traced fear paradigms were conducted. In the contextual memory recall test, the mouse was placed back to the shock chamber again for five minutes. After a short break in the home cages, the mouse was put into a novel chamber for the traced (tone-induced) fear memory recall test. The animal was allowed to explore freely for three minutes before the onset of the conditioned tone was played for 2 seconds (repeated seven times with random 1–3 min intervals between trace recall sessions). Freezing behaviors were scored in both retention tests. Throughout the procedures, animal behaviors were recorded simultaneously by videotaping which were synchronized with spike data collection. Freezing response, defined as absence of body movement except for respiration, was counted as the measurement of fear memory. We counted ‘freezing’ by observing the animal behaviors frame by frame based on the recorded video, the temporal resolution for the video is 30 frames per second and the absolute amount of time that the mice stayed in the continuous frozen state was summed and compared to the total amount of time.

### Characterization of CA1 unit responses to fear conditioning stimuli

To determine whether a recorded unit is responsive to a stimulus, we used the time of stimulus delivery as time zeros to calculate a peri-event histogram using a 100 msec window. The neural activities during two seconds before stimulus were used as the baseline to determine a set of confidence intervals of 80%, 95% to determine neural responsiveness. First, the 80% confidence intervals were used to determine the duration of response while there was any significant peak (positive response) or trough (negative response) happened within one second after stimulus. Second, if the peak or trough was out of the 95% confident intervals, and the duration was longer than 0.5 sec, then it is considered a significant neuronal response to the stimulus. To facilitate comparison between units that exhibit different increases/decreases over baseline activities, we examined normalized neural response:
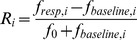
(1)


Here, 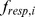 represents the average firing rate during detected duration, 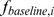 is the average firing rate during baseline before the 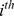 stimulus, and 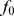 is the average population activity during rest states. This transformation allows for uniform quantification of the significant changes in firing patterns for units with both low- and high baseline firing rates.

### Hierarchical clustering

Hierarchical clustering methods were used to investigate the stimulus responses of the overall population of the simultaneously recorded CA1 units from both control and mutant mice. The procedure was described in our previously research [38], [40], [41]. This analysis was performed on the logarithm transformed neuronal response 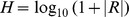. 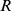 is an 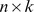 matrix represents the neuronal responses of 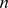 units during 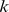 sampling points, 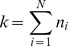 corresponding to the all repetitions of 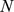 different stimulus while 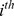 stimulus has 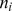 repetitions respectively, and 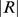 denotes the absolute value of neural responses. To build hierarchy, first, a Euclidean distance matrix was constructed to measure pair-wise distances between different units. An agglomerative hierarchical cluster tree was created from the individual unit, which means in the beginning of the process, each unit to different stimulus is in a cluster of itself. Then the clusters with the shortest distance were sequential combined into a larger cluster and the pair-wise distances between clusters were updated. After the cluster merging completed, a categorical sorting was applied to facilitate the visualization, in which units were sorted by the number of stimuli they responded. After sorting, the units responded to the most stimuli were put on the top, and the non-responsive units located at the bottom of the matrix.

### Dimensionality-reduction based statistical pattern projection methods

We then used Multiple Discriminant Analysis (MDA) methods to project the neural responses of hundred units corresponding to different stimuli into different classes in subspaces [38], [40], [41]. To account for transient changes that may occur immediately after CS or US stimuli, we computed responses by using firing frequencies in two 250 msec temporal windows around the delivery of the stimuli. Responses during baseline period were characterized by computing the average firing rates during time intervals preceding the CS or US stimuli. Thus, population neural responses to the stimulus CS or US were normalized and projected by MDA to form the CS or US cluster in subspace, in the meantime responses during baseline were projected to form the Rest cluster.

The matrix of the responses from hundreds of simultaneously recorded neurons corresponding to each event was used to compute the between-class scatter matrix and within-class matrix:
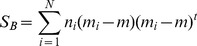
(2)

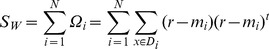
(3)


Here 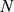 is the number of categories (different types of stimuli), 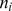 is the number of elements in each category (repetitions for each stimulus), 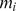 is the mean vector for each category, 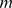 is the global mean vector from all categories and the symbol 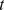 indicates the transpose operator, 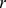 is the response of single neuron triggered by the 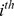 stimulus, 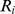 represents the set of population responses. Using these two matrices, the covariance matrix 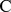 can be obtained by 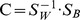. A set of at most 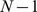 discriminant projection vectors can be determined by computing the eigenvalue decomposition of the covariance matrix 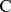.

For our data sets, the class covariance matrices 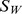
were non-invertible, which is a direct consequence of data under-sampling, since the number of recorded units is much more than the number of repeated trials. In practice, the matrix 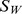
can be rendered invertible using a regularization technique which changes each class covariance matrices based on the following formula: 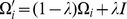, where 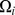
is the covariance matrix for the 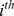 category, 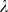 is a regularization parameter between 0 and 1, and 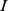 is the identity matrix**.** We determined the parameter 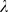
for each data set based on the optimization procedure we developed previously [41]; each particular choice was determined by the particular distributions within each data set [36], [41].

After computing the 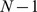 discriminant dimension, we can project the neural responses to low-dimensional encoding subspaces by transfer matrix, whose columns are the corresponding discriminant vectors and were sorted descend according to the eigenvalues. We can use the multivariate Gaussian distribution probability functions 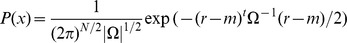 to fit the projections for each category. In addition, we used a 20-msec sliding temporal window to monitor the evolution of the population state throughout the duration of the experiment and to identify the occurrences of patterns similar to the ones produced by CS or US stimuli [36], [41].

For the tone-induced trace recall, it occurred in a novel environment which was different from the original training chamber. The global MDA analysis showed that tone-triggered responses during both learning and the recall test could still form a single CS cluster that was well separated from other events, indicating that major information carried by the tones during training and the recall was similar despite the contextual difference. However, the contextual difference can be further analyzed by using a second-step MDA analysis under which the CS ensemble trajectories elicited during recall can be separated from the CS ensemble trajectories during training [36].

### Statistical criteria for distinguishing trajectory types in MDA subspaces

To determine the individual identity of ensemble trajectories during both learning and retention tests, two measures were developed: 1) 4σ around cluster center was defined as the area close to the cluster in MDA subspaces, 2) since the distributions of the distance between the whole trajectories over the given trial and the cluster followed the Gaussian distribution, 2σ of the mean distance was used as a boundary for the trace candidate. The first measure provided global control across multiple trials while the second measure took the trial variability into consideration. A trace candidate should be close to the CS or US cluster, and at the same time far away from the Rest cluster. Specially, to quantify that, first, the trajectory could be determined close to the CS or US cluster, if the trace reached within 4σ from the cluster center, or if the distance between the trace candidate and the CS or US cluster reached less than 2σ below the mean distance of the Gaussian distribution (Bonferroni correction). Second, to be far away from the Rest, the farthest turning points on the trace candidate should be at least 4σ from the Rest cluster center, or the distance between the trace candidate and the Rest cluster should reach 2σ above the mean distance to the Rest (Bonferroni correction). Finally, movement directions of ensemble trajectories to the CS/US cluster and to the Rest cluster were visually confirmed by rotating the clusters in 3-D plots. By applying the above criteria, trace candidate were determined. If a trace reached CS cluster and then moved to US cluster, it was determined as a CS-to-US associative trace. If a trace reached US cluster and then to CS cluster, it was determined as a US-to-CS associative trace.

To examine the contributions of neurons to transient ensemble patterns, we shuffled spike trains of the perspective units and then performed MDA projections for comparison with that of trajectories obtained from original spike trains prior to shuffle [40]. Perspective units could be the units responsive to memory traces or non-responsive units as a comparison. Surrogate spike trains of perspective units were generated by randomizing spike time, while maintaining their original overall firing rates. It is expected that the information contained in original spike train like ISIs would be abolished by the shuffling process. The same 250 msec-moving window method was then applied on surrogate spike trains for MDA projections while using Identity matrix obtained from original MDA training on the global datasets. To avoid the effects of numbers of shuffled units on MDA patterns, we matched the number of perspective units, specifically the numbers of non-responsive units with the numbers of responsive units for spike train shuffling of each type of ensemble traces. Statistical criteria for defining trajectory types in MDA subspaces as described above were then applied.

## Supporting Information

Sound S1 **“Pavlovian memory symphony” of recall patterns of fear memory traces from the CA1 of the hippocampus of a wild-type mouse.** The audio file reflects the memory patterns generated from the CA1 of the hippocampus of a wild-type mouse (mouse #4 in Figure 12A). To convert the recall patterns of fear memory traces into “Pavlovian memory symphony”, four distinct types of fear memory traces retrieved during the five-minutes contextual recall session are represented by four different frequency tones (notes). Each tenor C, E, G and a soprano C note correspond to a simple CS trace, a simple US trace, a CS-to-US associative trace, and a US-to-CS associative trace, respectively. The audio clip of this 5-minute “Pavlovian memory symphony” is time compressed by a factor of four (into a 1.25-minute clip).
(WAV)

Sound S2 **“Pavlovian memory symphony” of recall patterns of fear memory traces from the CA1 of the hippocampus of a mutant mouse.** The audio file reflects the memory patterns generated from the CA1 of the hippocampus of a mutant mouse (knockout mouse #3 in Figure 12B). The conversion method is the same as Sound S1.
(WAV)

## References

[1] Hebb DO (1949) The Organization of Behavior. New York: Wiley.

[2] Andersen P (2003) A prelude to long-term potentiation. Philosophical transactions of the Royal Society of London Series B, Biological sciences 358: 613–615. 12740103 10.1098/rstb.2002.1232 PMC1693144

[3] Frey S, Frey JU (2008) 'Synaptic tagging' and 'cross-tagging' and related associative reinforcement processes of functional plasticity as the cellular basis for memory formation. Prog Brain Res 169: 117–143. 18394471 10.1016/S0079-6123(07)00007-6

[4] Neves G, Cooke SF, Bliss TV (2008) Synaptic plasticity, memory and the hippocampus: a neural network approach to causality. Nat Rev Neurosci 9: 65–75. 18094707 10.1038/nrn2303

[5] Tang YP, Shimizu E, Dube GR, Rampon C, Kerchner GA, et al (1999) Genetic enhancement of learning and memory in mice. Nature 401: 63–69. 10485705 10.1038/43432

[6] Tsien JZ, Huerta PT, Tonegawa S (1996) The essential role of hippocampal CA1 NMDA receptor-dependent synaptic plasticity in spatial memory. Cell 87: 1327–1338. 8980238 10.1016/s0092-8674(00)81827-9

[7] Cui Z, Wang H, Tan Y, Zaia KA, Zhang S, et al (2004) Inducible and reversible NR1 knockout reveals crucial role of the NMDA receptor in preserving remote memories in the brain. Neuron 41: 781–793. 15003177 10.1016/s0896-6273(04)00072-8

[8] Squire LR (1992) Memory and the hippocampus: a synthesis from findings with rats, monkeys, and humans. Psychol Rev 99: 195–231. 1594723 10.1037/0033-295x.99.2.195

[9] Thompson RF (2005) In search of memory traces. Annu Rev Psychol 56: 1–23. 15709927 10.1146/annurev.psych.56.091103.070239

[10] Lin L, Osan R, Tsien JZ (2006) Organizing principles of real-time memory encoding: neural clique assemblies and universal neural codes. Trends Neurosci 29: 48–57. 16325278 10.1016/j.tins.2005.11.004

[11] Tsien JZ (2007) The memory code. Sci Am 297: 52–59. 10.1038/scientificamerican0707-52 17695842

[12] Alivisatos AP, Chun M, Church GM, Greenspan RJ, Roukes ML, et al (2012) The brain activity map project and the challenge of functional connectomics. Neuron 74: 970–974. 22726828 10.1016/j.neuron.2012.06.006 PMC3597383

[13] Tsien JZ, Li M, Osan R, Chen G, Lin L, et al (2013) On initial Brain Activity Mapping of episodic and semantic memory code in the hippocampus. Neurobiol Learn Mem 105: 200–210. 23838072 10.1016/j.nlm.2013.06.019 PMC3769419

[14] O'Keefe J, Dostrovsky J (1971) The hippocampus as a spatial map. Preliminary evidence from unit activity in the freely-moving rat. Brain Res 34: 171–175. 5124915 10.1016/0006-8993(71)90358-1

[15] Wilson MA, McNaughton BL (1993) Dynamics of the hippocampal ensemble code for space. Science 261: 1055–1058. 8351520 10.1126/science.8351520

[16] Hampson RE, Pons TP, Stanford TR, Deadwyler SA (2004) Categorization in the monkey hippocampus: a possible mechanism for encoding information into memory. Proc Natl Acad Sci U S A 101: 3184–3189. 14978264 10.1073/pnas.0400162101 PMC365764

[17] Quiroga RQ, Reddy L, Kreiman G, Koch C, Fried I (2005) Invariant visual representation by single neurons in the human brain. Nature 435: 1102–1107. 15973409 10.1038/nature03687

[18] Smith DM, Mizumori SJ (2006) Hippocampal place cells, context, and episodic memory. Hippocampus 16: 716–729. 16897724 10.1002/hipo.20208

[19] Lin L, Chen G, Kuang H, Wang D, Tsien JZ (2007) Neural encoding of the concept of nest in the mouse brain. Proc Natl Acad Sci U S A 104: 6066–6071. 17389405 10.1073/pnas.0701106104 PMC1851617

[20] Buzsaki G, Moser EI (2013) Memory, navigation and theta rhythm in the hippocampal-entorhinal system. Nat Neurosci 16: 130–138. 23354386 10.1038/nn.3304 PMC4079500

[21] Naya Y, Suzuki WA (2011) Integrating what and when across the primate medial temporal lobe. Science 333: 773–776. 21817056 10.1126/science.1206773

[22] Clark RE, Zola S (1998) Trace eyeblink classical conditioning in the monkey: a nonsurgical method and behavioral analysis. Behav Neurosci 112: 1062–1068. 9829784 10.1037//0735-7044.112.5.1062

[23] Kim JJ, Jung MW (2006) Neural circuits and mechanisms involved in Pavlovian fear conditioning: a critical review. Neurosci Biobehav Rev 30: 188–202. 16120461 10.1016/j.neubiorev.2005.06.005 PMC4342048

[24] Maren S (2001) Neurobiology of Pavlovian fear conditioning. Annu Rev Neurosci 24: 897–931. 11520922 10.1146/annurev.neuro.24.1.897

[25] Chowdhury N, Quinn JJ, Fanselow MS (2005) Dorsal hippocampus involvement in trace fear conditioning with long, but not short, trace intervals in mice. Behav Neurosci 119: 1396–1402. 16300446 10.1037/0735-7044.119.5.1396

[26] McNally GP, Johansen JP, Blair HT (2011) Placing prediction into the fear circuit. Trends Neurosci 34: 283–292. 21549434 10.1016/j.tins.2011.03.005 PMC4245078

[27] Bangasser DA, Waxler DE, Santollo J, Shors TJ (2006) Trace conditioning and the hippocampus: the importance of contiguity. J Neurosci 26: 8702–8706. 16928858 10.1523/JNEUROSCI.1742-06.2006 PMC3289537

[28] Knight DC, Cheng DT, Smith CN, Stein EA, Helmstetter FJ (2004) Neural substrates mediating human delay and trace fear conditioning. J Neurosci 24: 218–228. 14715954 10.1523/JNEUROSCI.0433-03.2004 PMC6729570

[29] McEchron MD, Bouwmeester H, Tseng W, Weiss C, Disterhoft JF (1998) Hippocampectomy disrupts auditory trace fear conditioning and contextual fear conditioning in the rat. Hippocampus 8: 638–646. 9882021 10.1002/(SICI)1098-1063(1998)8:6<638::AID-HIPO6>3.0.CO;2-Q

[30] Jovanovic T, Norrholm SD, Blanding NQ, Phifer JE, Weiss T, et al (2010) Fear potentiation is associated with hypothalamic-pituitary-adrenal axis function in PTSD. Psychoneuroendocrinology 35: 846–857. 20036466 10.1016/j.psyneuen.2009.11.009 PMC2875386

[31] Davis M (1986) Pharmacological and anatomical analysis of fear conditioning using the fear-potentiated startle paradigm. Behav Neurosci 100: 814–824. 3545257 10.1037//0735-7044.100.6.814

[32] Davis M (2001) Fear-potentiated startle in rats. Curr Protoc Neurosci Chapter 8: Unit 8 11A. 10.1002/0471142301.ns0811as14 18428539

[33] Liu J, Wei W, Kuang H, Zhao F, Tsien JZ (2013) Changes in heart rate variability are associated with expression of short-term and long-term contextual and cued fear memories. PLoS ONE 8: e63590. 23667644 10.1371/journal.pone.0063590 PMC3646801

[34] Misane I, Tovote P, Meyer M, Spiess J, Ogren SO, et al (2005) Time-dependent involvement of the dorsal hippocampus in trace fear conditioning in mice. Hippocampus 15: 418–426. 15669102 10.1002/hipo.20067

[35] Raybuck JD, Lattal KM (2011) Double dissociation of amygdala and hippocampal contributions to trace and delay fear conditioning. PLoS ONE 6: e15982. 21283812 10.1371/journal.pone.0015982 PMC3023765

[36] Chen G, Wang LP, Tsien JZ (2009) Neural population-level memory traces in the mouse hippocampus. PLoS One 4: e8256. 20016843 10.1371/journal.pone.0008256 PMC2788416

[37] Kuang H, Lin L, Tsien JZ (2010) Temporal dynamics of distinct CA1 cell populations during unconscious state induced by ketamine. PLoS ONE 5: e15209. 21165147 10.1371/journal.pone.0015209 PMC2999569

[38] Lin L, Osan R, Shoham S, Jin W, Zuo W, et al (2005) Identification of network-level coding units for real-time representation of episodic experiences in the hippocampus. Proc Natl Acad Sci U S A 102: 6125–6130. 15833817 10.1073/pnas.0408233102 PMC1087910

[39] Lin L, Chen G, Xie K, Zaia KA, Zhang S, et al (2006) Large-scale neural ensemble recording in the brains of freely behaving mice. J Neurosci Methods 155: 28–38. 16554093 10.1016/j.jneumeth.2005.12.032

[40] Osan R, Chen G, Feng R, Tsien JZ (2011) Differential Consolidation and Pattern Reverberations within Episodic Cell Assemblies in the Mouse Hippocampus. PLoS ONE 6: e16507. 21347227 10.1371/journal.pone.0016507 PMC3039647

[41] Osan R, Zhu L, Shoham S, Tsien JZ (2007) Subspace projection approaches to classification and visualization of neural network-level encoding patterns. PLoS ONE 2: e404. 17476326 10.1371/journal.pone.0000404 PMC1852331

[42] McEchron MD, Tseng W, Disterhoft JF (2003) Single neurons in CA1 hippocampus encode trace interval duration during trace heart rate (fear) conditioning in rabbit. J Neurosci 23: 1535–1547. 12598642 10.1523/JNEUROSCI.23-04-01535.2003 PMC6742268

[43] Davis M (2011) NMDA receptors and fear extinction: implications for cognitive behavioral therapy. Dialogues Clin Neurosci 13: 463–474. 22275851 10.31887/DCNS.2011.13.4/mdavis PMC3263393

[44] Izquierdo I, Medina JH (1997) Memory formation: the sequence of biochemical events in the hippocampus and its connection to activity in other brain structures. Neurobiol Learn Mem 68: 285–316. 9398590 10.1006/nlme.1997.3799

[45] Matus-Amat P, Higgins EA, Sprunger D, Wright-Hardesty K, Rudy JW (2007) The role of dorsal hippocampus and basolateral amygdala NMDA receptors in the acquisition and retrieval of context and contextual fear memories. Behav Neurosci 121: 721–731. 17663597 10.1037/0735-7044.121.4.721

[46] Quinn JJ, Loya F, Ma QD, Fanselow MS (2005) Dorsal hippocampus NMDA receptors differentially mediate trace and contextual fear conditioning. Hippocampus 15: 665–674. 15959918 10.1002/hipo.20088

[47] Moita MA, Rosis S, Zhou Y, LeDoux JE, Blair HT (2004) Putting fear in its place: remapping of hippocampal place cells during fear conditioning. J Neurosci 24: 7015–7023. 15295037 10.1523/JNEUROSCI.5492-03.2004 PMC6729593

[48] Disterhoft JF, Coulter DA, Alkon DL (1986) Conditioning-specific membrane changes of rabbit hippocampal neurons measured in vitro. Proc Natl Acad Sci U S A 83: 2733–2737. 3458232 10.1073/pnas.83.8.2733 PMC323374

[49] Descalzi G, Li XY, Chen T, Mercaldo V, Koga K, et al (2012) Rapid synaptic potentiation within the anterior cingulate cortex mediates trace fear learning. Mol Brain 5: 6. 22304729 10.1186/1756-6606-5-6 PMC3395850

[50] Song C, Detert JA, Sehgal M, Moyer JR Jr (2012) Trace fear conditioning enhances synaptic and intrinsic plasticity in rat hippocampus. J Neurophysiol 107: 3397–3408. 22442572 10.1152/jn.00692.2011

[51] Shimizu E, Tang YP, Rampon C, Tsien JZ (2000) NMDA receptor-dependent synaptic reinforcement as a crucial process for memory consolidation. Science 290: 1170–1174. 11073458 10.1126/science.290.5494.1170

[52] Czerniawski J, Ree F, Chia C, Otto T (2012) Dorsal versus ventral hippocampal contributions to trace and contextual conditioning: differential effects of regionally selective NMDA receptor antagonism on acquisition and expression. Hippocampus 22: 1528–1539. 22180082 10.1002/hipo.20992

[53] Walker DL, Davis M (2008) Amygdala infusions of an NR2B-selective or an NR2A-preferring NMDA receptor antagonist differentially influence fear conditioning and expression in the fear-potentiated startle test. Learn Mem 15: 67–74. 18230675 10.1101/lm.798908 PMC2216678

[54] Kim JJ, Clark RE, Thompson RF (1995) Hippocampectomy impairs the memory of recently, but not remotely, acquired trace eyeblink conditioned responses. Behav Neurosci 109: 195–203. 7619310 10.1037//0735-7044.109.2.195

[55] Kim JJ, Fanselow MS (1992) Modality-specific retrograde amnesia of fear. Science 256: 675–677. 1585183 10.1126/science.1585183

[56] Quinn JJ, Oommen SS, Morrison GE, Fanselow MS (2002) Post-training excitotoxic lesions of the dorsal hippocampus attenuate forward trace, backward trace, and delay fear conditioning in a temporally specific manner. Hippocampus 12: 495–504. 12201634 10.1002/hipo.10029

[57] Biedenkapp JC, Rudy JW (2007) Context preexposure prevents forgetting of a contextual fear memory: implication for regional changes in brain activation patterns associated with recent and remote memory tests. Learn Mem 14: 200–203. 17351145 10.1101/lm.499407 PMC2519802

[58] Sara SJ (2010) Reactivation, retrieval, replay and reconsolidation in and out of sleep: connecting the dots. Front Behav Neurosci 4: 185. 21179586 10.3389/fnbeh.2010.00185 PMC3004439

[59] Wang SH, de Oliveira Alvares L, Nader K (2009) Cellular and systems mechanisms of memory strength as a constraint on auditory fear reconsolidation. Nat Neurosci 12: 905–912. 19543280 10.1038/nn.2350

[60] Pang MH, Kim NS, Kim IH, Kim H, Kim HT, et al (2010) Cholinergic transmission in the dorsal hippocampus modulates trace but not delay fear conditioning. Neurobiol Learn Mem 94: 206–213. 20685338 10.1016/j.nlm.2010.05.008

[61] Bang SJ, Brown TH (2009) Muscarinic receptors in perirhinal cortex control trace conditioning. J Neurosci 29: 4346–4350. 19357262 10.1523/JNEUROSCI.0069-09.2009 PMC2692993

[62] Navakkode S, Sajikumar S, Sacktor TC, Frey JU (2010) Protein kinase Mzeta is essential for the induction and maintenance of dopamine-induced long-term potentiation in apical CA1 dendrites. Learn Mem 17: 605–611. 21084457 10.1101/lm.1991910 PMC2998336

[63] Korz V, Frey JU (2007) Hormonal and monoamine signaling during reinforcement of hippocampal long-term potentiation and memory retrieval. Learn Mem 14: 160–166. 17351139 10.1101/lm.459807 PMC1838557

[64] Lisman JE, Grace AA (2005) The hippocampal-VTA loop: controlling the entry of information into long-term memory. Neuron 46: 703–713. 15924857 10.1016/j.neuron.2005.05.002

[65] Manns JR, Clark RE, Squire LR (2000) Awareness predicts the magnitude of single-cue trace eyeblink conditioning. Hippocampus 10: 181–186. 10791840 10.1002/(SICI)1098-1063(2000)10:2<181::AID-HIPO7>3.0.CO;2-V

[66] Gilmartin MR, Kwapis JL, Helmstetter FJ (2012) Trace and contextual fear conditioning are impaired following unilateral microinjection of muscimol in the ventral hippocampus or amygdala, but not the medial prefrontal cortex. Neurobiol Learn Mem 97: 452–464. 22469748 10.1016/j.nlm.2012.03.009 PMC3358523

[67] Hylin MJ, Orsi SA, Moore AN, Dash PK (2013) Disruption of the perineuronal net in the hippocampus or medial prefrontal cortex impairs fear conditioning. Learn Mem 20: 267–273. 23592037 10.1101/lm.030197.112 PMC3630486

[68] Hyman JM, Zilli EA, Paley AM, Hasselmo ME (2010) Working Memory Performance Correlates with Prefrontal-Hippocampal Theta Interactions but not with Prefrontal Neuron Firing Rates. Front Integr Neurosci 4: 2. 20431726 10.3389/neuro.07.002.2010 PMC2861479

[69] Steenland HW, Li X-Y, Zhuo M (2012) Predicting aversive events and terminating fear in the mouse anterior cingulate cortex during trace fear conditioning. The Journal of neuroscience : the official journal of the Society for Neuroscience 32: 1082–1095. 22262906 10.1523/JNEUROSCI.5566-11.2012 PMC6621139

[70] Wang LP, Li F, Wang D, Xie K, Shen X, et al (2011) NMDA Receptors in Dopaminergic Neurons Are Crucial for Habit Learning. Neuron 72: 1055–1066. 22196339 10.1016/j.neuron.2011.10.019 PMC3246213

[71] Schmitzer-Torbert N, Jackson J, Henze D, Harris K, Redish AD (2005) Quantitative measures of cluster quality for use in extracellular recordings. Neuroscience 131: 1–11. 15680687 10.1016/j.neuroscience.2004.09.066

